# Entscheidungsfindung und Einfluss bei Fridays for Future – Zur Rolle von Basisdemokratie, Hierarchien und Expertise

**DOI:** 10.1007/s41358-023-00341-x

**Published:** 2023-02-02

**Authors:** Witold Mucha, Anna Soßdorf, Laura Ferschinger, Viktor Burgi, Philipp Krach

**Affiliations:** Heinrich-Heine-Universität Düsseldorf, Düsseldorf, Deutschland

**Keywords:** Fridays for Future, Entscheidungen, Einfluss, Citizen Science, Soziale Bewegungen, Klimabewegungen, Fridays for Future, Decisions, Influence, Citizen science, Social movements, Climate movements

## Abstract

Im Unterschied zur bisherigen Forschung über Fridays for Future, die sich vornehmlich mit der Genese, Mobilisierung und dem Framing der sozialen Bewegung beschäftigt, stehen deren interne Entscheidungsprozesse im Mittelpunkt des Aufsatzes: Wie werden Entscheidungen in der dezentral organisierten Bewegung getroffen und welche Faktoren haben dabei besonderen Einfluss? Auf Basis eines Mehrmethodendesigns, das sich Beobachtungen, Interviews und einer Online-Umfrage bedient, kommt die Studie zu drei Ergebnissen. Erstens bewegen sich Entscheidungsdynamiken insbesondere auf Bundesebene im Spannungsfeld zwischen basisdemokratischen Grundprinzipien auf der einen und dem Primat effizienter Prozesse auf der anderen Seite. Zweitens herrschen informelle Hierarchien innerhalb der verschiedenen Ebenen vor, die sowohl positiv als auch negativ von den Mitgliedern bewertet werden. Drittens nimmt der Einfluss auf Entscheidungen zu je erfahrener, kompetenter und besser vernetzt Aktivist:innen sind. Die Implikationen der Ergebnisse für anknüpfende Forschung im Bereich der Theorien der Sozialen Bewegungen sind zweierlei. Zum einen wird deutlich, dass eine Organisation wie Fridays for Future unabhängig der dezentralen Autonomie ihrer über 500 Ortsgruppen auch nach über drei Jahren seit ihrer Gründung entscheidungs- und mobilisierungsfähig geblieben ist. Im Vergleich zu anderen sozialen Bewegungen beweist Fridays for Future hinsichtlich ihres Wirkungsradius und ihrer Mitgliederstärke somit eine erstaunliche Resilienz. Zum anderen zeigen die Ergebnisse, dass es trotz einer beachtlichen Formalisierung und Differenzierung auf Seiten der Basis durchaus Unmut über Machtasymmetrien gegenüber den Entscheidungen auf Bundesebene gibt. Die Ergebnisse fußen auf einem Citizen-Science-Projekt, das etablierte Aktivist:innen der Bewegung mit Wissenschaftler:innen zusammenbrachte. Die Kooperation ermöglichte den exklusiven Zugang zu den Entscheidungsforen der Kernmitglieder.

**Zusatzmaterial online:**

Zusätzliche Informationen sind in der Online-Version dieses Artikels (10.1007/s41358-023-00341-x) enthalten.

## Abstract (en)

The focus of the article is the internal decision-making processes at Fridays for Future: How are decisions made in the decentral organized Movement and what are the most influential factors in that regard? This is in contrast to the majority of studies that have predominantly looked into the genesis, mobilization and framing aspects of the Movement. Based on a multi-method design (i.e. observations, interviews, online survey), the findings are threefold. First, particularly at federal level decision-making processes are characterized by balancing between grassroots democracy on the one hand and the need for efficiency on the other. Second, there are elements of informal hierarchy at different levels of the Movement. Members evaluate those hierarchies both positively and negatively. Third, the more experience, competence, and networks members have the stronger their leverage is on decisions. The results hold two implications with regard to further research in Social Movement Theory. On the one hand, the analysis shows that Fridays for Future is still capable of making decisions and mobilizing its members—even after three years since it was founded and regardless of the autonomy of its more than 500 local groups. This demonstrates its range of impact as well as striking resilience compared to other contemporary Social Movements. On the other hand, the findings reveal some discontent among locally based members with regard to power asymmetries related to decisions made on federal level. The data presented in this article is based on a citizen science project that brought activists and scholars together. The cooperation facilitated the exclusive access to the internal decision-making processes at Fridays for Future.

## Einleitung

Das Wirken von Fridays For Future (FFF) wird von Seiten der Öffentlichkeit und den Sozialwissenschaften seit dem ersten Schulstreik von Greta Thunberg im August 2018 aufmerksam verfolgt (Rucht 2019a, b; Sommer et al. 2019; Wahlström et al. 2019; de Moor et al. 2020). In der jüngeren Fachliteratur wurde besonderes Augenmerk erstens auf die Genese, die Mobilisierung und das Framing der sozialen Bewegung gelegt (Brünker et al. 2019; Berker und Pollex 2021; Huth 2020; Raisch und Zohlnhöfer 2020; Fopp et al. 2021; Holmberg 2021; Huttunen und Albrecht 2021; von Zabern und Tulloch 2021). Jenen Studien nach handelt es sich bei den Protestler:innen um überwiegend weibliche, aus der höher gebildeten Mittelschicht stammende und progressiv eingestellte Jugendliche (Haunss und Sommer 2020). Neben dem hohen Frauenanteil und der außergewöhnlich jungen Altersstruktur unterscheidet sich FFF von anderen sozialen Bewegungen auch durch ihre Reichweite und Fähigkeit, junge Menschen zur Partizipation zu mobilisieren (Hurrelmann und Albrecht 2020). Die Motivation von Aktivist:innen an FFF-Protesten teilzunehmen bildete einen zweiten Schwerpunkt in der Debatte, in der Konsens herrscht, dass sowohl soziale als auch persönliche Normen identitätsstiftend seien (Daniel et al. 2020; Huttunen 2021; Wallis und Loy 2021). Vor dem Hintergrund der Covid-19-Pandemie beschäftigten sich Studien drittens mit dem Umgang von FFF mit den durch die Kontaktbeschränkungen einhergehenden Herausforderungen. Diese kommen zu dem Schluss, dass sich FFF den veränderten Rahmenbedingungen anpasst, indem die Aktivist:innen ihre Protestformen in den digitalen Raum verlagern und auf ihre bereits zuvor etablierten Strukturen und Erfahrungen der online-basierten Kommunikations‑, Organisations‑, Mobilisierungs- und Protestformen zurückgreifen (Mucha et al. 2020; Haßler et al. 2021; Hunger und Hutter 2021; Sorce und Dumitrica 2021).

Trotz dieser Fülle von Studien weist die Fachliteratur eine Forschungslücke im Hinblick auf die internen Entscheidungsprozesse von FFF auf. Die zentralen Fragen lauten: Wie werden Entscheidungen in einer dezentral organisierten Bewegung wie FFF getroffen und welche Faktoren haben dabei besonderen Einfluss? Vereinzelt werden diese Fragen in Bezug auf FFF zwar gestellt, allerdings fehlt es den jeweiligen Arbeiten an einer breiten empirischen Basis, die nicht nur einzelne der insgesamt ca. 650 Ortsgruppen (OGs), sondern die gesamte Bewegung in Deutschland vergleichend in den Blick nimmt (Döninghaus et al. 2020; Marquardt 2020; Rucht und Rink 2020; Soler-i-Martí et al. 2020; Wandzilak 2020; Della Porta und Portos 2021). Döninghaus et al. (2020) stellten beispielsweise fest, dass im Mittelpunkt von FFF-Entscheidungen eine Wertorientierung um Klimaschutz und Klimagerechtigkeit stehe, nach der die Bewegung ihr kollektives Handeln ausrichte. Der Entscheidungsprozess beruhe demnach auf zwei Arten der Konsensfindung, dem anerkannten sowie dem Grundkonsens. Beide zeichneten sich dadurch aus, dass explizit nach Gegenmeinungen gefragt würde. Der Grundkonsens biete zudem in der Diskussion Möglichkeiten, konträre Ansichten darzulegen. Die Beteiligten bemühten sich zwar um eine gleichberechtigte Kommunikation, dennoch käme es zur Herausbildung von Hierarchien (Döninghaus et al. 2020).

Im Unterschied zu den von Döninghaus et al. (2020) auf einer teilnehmenden Beobachtung von insgesamt vier Sitzungen von zwei Ortsgruppen in Bremen und Bremerhaven basierenden Daten, fußt der vorliegende Beitrag auf Ergebnissen eines Citizen-Science-Projektes (2020–2021), das FFF-Aktivist:innen aus Nordrhein-Westfalen in den Forschungsprozess einband. Mit ihrer Hilfe war es möglich, einen exklusiven Zugang zu den Entscheidungsforen der Kernmitglieder herzustellen, so dass die Entscheidungsprozesse und die gelebten Routinen sowohl auf Bundes- als auch auf Ortsgruppenebene untersucht werden konnten. Die Erhebung wurde von dem übergeordneten Forschungsinteresse geleitet, wie Entscheidungen innerhalb von FFF getroffen werden und welche spezifischen Faktoren Einfluss auf die Entscheidungsfindung ausüben. Das Mehrmethodendesign fußte dabei auf drei Zugängen. Erstens fanden sieben teilnehmende Beobachtungen in Ortsgruppen und Delegiertenkonferenzen statt. Zweitens wurden zwölf Interviews mit etablierten Aktivist:innen geführt und drittens wurde eine bundesweite Online-Umfrage geschaltet, an der 507 Aktivist:innen teilnahmen.

Der Citizen Science-Ansatz bedingt den Fokus auf FFF in Deutschland. Der Anspruch besteht nicht darin, Aussagen über FFF generell zu treffen. Allein die (Bevölkerungs‑)Größe, Geschichte Deutschlands (z. B. Wiedervereinigung) oder zivilgesellschaftliche Tradition (z. B. Anti-Atomkraft-Bewegung) können Charakteristika in Bezug auf Entscheidungsprozesse bedienen, die einen Vergleich mit Bewegungen beispielsweise in Finnland oder Italien erschweren. In anderen Worten ist FFF in Deutschland aufgrund der bundesweiten Größe der Organisation von Natur aus weiter institutionalisiert. Entsprechend herrscht ein anderer Benchmark beispielsweise im Vergleich zu FFF in Spanien vor. Auch innerhalb der deutschen FFF-Bewegungslandschaft und ihren 650 OGs ist von lokaler Heterogenität auszugehen (vgl. Kap. 4). Bei der vorliegenden Studie handelt es sich demnach um die Analyse einer typischen eher institutionalisierten FFF-Version. Nichtsdestotrotz ist es sinnvoll, sich mit dem Fall von FFF in Deutschland auseinanderzusetzen, weil es sich dabei um eine etablierte FFF-Bewegung handelt, die anknüpfenden Studien Aufschluss über die Wirkungskraft von Ableger:innen in anderen Ländern geben können. Ferner liegen im Sinne explorativer Forschung keine vergleichbaren Daten zu Entscheidungsprozessen von FFF jenseits von Deutschland vor. Die wenigen komparativen Studien beschäftigen sich in erster Linie mit der soziokulturellen Zusammensetzung (Neuber et al. 2020; Della Porta und Portos 2021; Giugni und Grasso 2021) oder dem Framing von Klimaaktivist:innen (Kern und Opitz 2021; Svensson und Wahlström 2021; Zamponi et al. 2022). Darüber hinaus ist die Fallstudie aus einer Generationsperspektive relevant. Es ist davon auszugehen, dass die wenige Erfahrung im politischen Aktivismus und die homogene Altersverteilung der jungen Bewegung für die Analyse interner Entscheidungsprozesse von Bedeutung sein können: „[Young] adults tend to pursue horizontal strategies and targets rather than top-down ones (Coe 2021, S. 19).“ Das gilt sowohl für die Herausbildung von Hierarchien als auch für die interne Kohäsion. Beispielsweise kann eine junge Kohorte die Stabilität einer Bewegung gefährden, wenn die Aktivist:innen zuvor keine langfristigen Kompromisse mit der Gruppe eingegangen sind (Whittier 1997).

Im Rahmen des Projekts wurden drei wesentliche Erkenntnisse gewonnen. Erstens bewegen sich Entscheidungsdynamiken insbesondere auf Bundesebene im Spannungsfeld zwischen basisdemokratischen Grundprinzipien auf der einen und dem Primat effizienter Prozesse auf der anderen Seite. Zweitens herrschen informelle Hierarchien innerhalb der verschiedenen Ebenen vor, die sowohl positiv als auch negativ von den Mitgliedern bewertet werden. Drittens nimmt der Einfluss auf Entscheidungen zu, je erfahrener, kompetenter und besser vernetzt Aktivist:innen sind. Die Implikationen der Ergebnisse für anknüpfende Forschung im Bereich der Theorien der Sozialen Bewegungen sind zweierlei. Zum einen wird deutlich, dass eine Organisation wie FFF, unabhängig von der dezentralen Autonomie ihrer 650 OGs, auch nach drei Jahren seit ihrer Gründung entscheidungs- und mobilisierungsfähig geblieben ist. Im Vergleich zu anderen sozialen Bewegungen der jüngeren Vergangenheit wie beispielsweise Occupy Wallstreet (OW) oder Extinction Rebellion (ER) beweist FFF hinsichtlich ihres Wirkungsradius und ihrer Mitgliederstärke somit eine erstaunliche Resilienz (Roberts 2012; Welch 2018; Mucha et al. 2020; De Moor et al. 2021; Fopp et al. 2021; Friberg 2021). Zum anderen zeigen die Ergebnisse jedoch auch, dass es trotz einer beachtlichen Formalisierung und Differenzierung auf Seiten der Basis durchaus Unmut über Machtasymmetrien gegenüber den Entscheidungen auf Bundesebene gibt.

Der Artikel ist in fünf Teile gegliedert. Im Anschluss an die Einleitung wird das theoretische Fundament des Beitrags begründet, indem Entscheidungstheorien aus der Organisationssoziologie, Bewegungsforschung und Konzepten der Deliberativen Demokratie eingeführt werden. Auf dieser Basis wird das methodologische Gerüst der Analyse im Hinblick auf die teilnehmenden Beobachtungen, die Interviews und die Umfragen vorgestellt. Die empirischen Ergebnisse werden im vierten Abschnitt dargestellt. Die Implikationen der Ergebnisse werden schließlich im Fazit diskutiert.

## Soziale Bewegungen und Entscheidungstheorien

Grundsätzlich entspricht FFF den klassischen definitorischen Merkmalen einer sozialen Bewegung. Die Aktivist:innen bilden ein Netzwerk bestehend aus Organisationen und Individuen, das auf Basis ihrer geteilten kollektiven Identität mit Hilfe von überwiegend nicht-institutionalisierten Methoden versucht, politischen Wandel herbeizuführen (Diani 1992; Della Porta und Diani 2020). Gleichwohl sei je nach Definition insofern zu differenzieren, dass FFF trotz seiner OGs, bundesweiten Arbeitsgruppen (AGs) und internationalen Schwesterallianzen eine Organisation der Klimagerechtigkeitsbewegung ist. Auch wenn FFF netzwerkförmig organisiert ist, wird sie über gemeinsame Ziele, Vernetzung und gemeinsame Aktivitäten, zusammen mit anderen Organisationen, als Teil der Klimagerechtigkeitsbewegung integriert (Sommer et al. 2019; Ruser 2020; Boscheinen und Bortfeldt 2021). Jenseits klassischer Theorien, die sich mit relativer Deprivation (Gurr 1970; Gurney und Tierney 1982), der Mobilisierung von Ressourcen (McCarthy und Zald 1977; Edwards und McCarthy 2004), Gelegenheitsstrukturen (Eisinger 1973; Kitschelt 1986), oder dem Framing (Snow et al. 1986; Benford und Snow 2000) beschäftigen, werden die Unterschiede sozialer Bewegungen entlang von drei Dimensionen behandelt: hinsichtlich ihrer Struktur, ihrer Institutionalisierungsgrade, und im Hinblick auf ihren Charakter als Konsens- oder Konfliktbewegung. Die Struktur sozialer Bewegungen wird unterschieden zwischen traditionell formalen Organisationen auf der einen und einem dezentralisierten Netzwerk von autonomen Gruppen auf der anderen Seite (Freeman 1979; Stickler 2005). Der Institutionalisierungsgrad sozialer Bewegungen wird anhand der zwei intra-organisationalen Prozesse der Differenzierung und Formalisierung diskutiert (Rucht et al. 1997; Pfister 2019). Zuletzt geht es bei der Unterscheidung zwischen Konsens- oder Konfliktbewegung um die öffentliche Akzeptanz von sozialen Bewegungen (McCarthy und Wolfson 1992; Della Porta 2009a).

Im Lichte der Vielfalt von sozialen Bewegungen besteht in der Fachdebatte eine Forschungslücke im Hinblick auf die internen Entscheidungsprozesse. Theoretische Anknüpfungspunkte lassen sich in Arbeiten zur Bewegungsforschung, zur deliberativen Demokratie und zur Organisationssoziologie finden (Sutherland et al. 2014; Bosse 2019). Der erste Strang konzentriert sich auf die Entscheidungsstrukturen bzw. die formalen Muster, nach denen kollektive Entscheidungsprozesse in sozialen Bewegungen ablaufen (Della Porta 2005, 2009b; Daphi 2019). In diesem Zusammenhang werden ferner funktionale Charakteristika basisdemokratischer Entscheidungen behandelt (Maeckelbergh 2009; Della Porta und Rucht 2013; Polletta und Jasper 2001, Polletta 2002, 2013; Della Porta und Doerr 2018). In der Fachdebatte zur deliberativen Demokratie bilden vornehmlich die Diskussions- und Aushandlungsprozesse den Schwerpunkt der Forschung. Das Gros der Arbeiten untersucht, inwiefern zivilgesellschaftliche Akteur:innen im Rahmen öffentlich diskursiver Prozesse in der Lage sind, demokratische Prinzipien und Verfahren gesamtgesellschaftlich zu verbessern (Maeckelbergh 2009; Della Porta 2015; Rowell 2015; Tanasoca 2020). Gleichwohl beschäftigen sich nur wenige Studien mit Entscheidungsprozessen auf Mikroebene (Ercan und Hendriks 2013; Lafont 2015; Kersting 2021), die für die hiesige Analyse der Dynamiken von FFF auf Ortsgruppen- und Delegiertenebene von Bedeutung sind. Abhilfe schaffen dabei Erkenntnisse der Organisationssoziologie (Wilz 2010; Kühl 2015; Nienhüser 2016; Preisendörfer 2016). Die Unterschiede der Betrachtungsweise in der Disziplin liegen zum einen in dem Interesse an Entscheidungsfähigkeiten entweder von einzelnen oder von kollektiven Akteur:innen. Zum anderen beschäftigen sich Forscher:innen mit unterschiedlichen Entscheidungsmodellen, die auf Basis normativer, deskriptiver oder präskriptiver Handlungsmuster argumentieren (Kirchler und Schrott 2003; Bosse 2019).

Im Hinblick auf kollektive Entscheidungsprozesse wird in der Organisationssoziologie insbesondere der mikropolitische Ansatz der Carnegie School diskutiert, der von begrenzt rationalen Akteur:innen ausgeht. Macht kollektiver Akteur:innen ergäbe sich in diesem Zusammenhang aus der Fähigkeit, Ungewissheit für sich zu nutzen bzw. über Wissensvorsprünge gegenüber anderen zu verfügen (Cyert und March 2005; Crozier und Friedberg 1979). In Anlehnung an Felsch (2010) wird Macht dabei nicht als Eigenschaft eines, sondern als Beziehung zwischen Akteur:innen verstanden. Konkret handelt es sich um Entscheidungen, die gemeinsam im Kollektiv und nicht von Einzelpersonen getroffen werden (Neuberger 1995; Felsch 2010; Wilz 2010). Zur Analyse der Macht bzw. des Einflusses in einer dezentral organisierten Bewegung wie FFF bieten sich insbesondere theoretische Erkenntnisse aus der Bewegungsforschung und der Elitensoziologie an (Freeman 1979, 2000; Sutherland et al. 2014). Freeman (2000) nach zu urteilen entwickeln Gruppen unweigerlich spezifische Strukturen zur Organisation der alltäglichen kollektiven Arbeit – seien es Entscheidungen zum Ablauf von Treffen, der Dauer, des Ortes oder generell der Entscheidungsmodi. Im Zuge der jeweiligen lokalen Dynamiken werden einzelnen Mitgliedern automatisch Rollen und Funktionen zugewiesen, auch wenn formell Konsens über Gleichberechtigung vorherrscht: „At any small group meeting anyone with a sharp eye and an acute ear can tell who is influencing whom. (…) They are nuances of interaction, not pre-written scripts. But they are discernible, and they do have their effect“ (Freeman 2000, S. 2).

### Informelle Hierarchien

Sutherland et al. (2014) argumentieren im Hinblick auf jene Zuweisung von Rollen und Funktionen, dass die formellen oder informellen Machthierarchien sowohl die finalen Entscheidungen als auch deren Alternativen beeinflussen, indem Meinungsführer:innen vorab bestimmen, welche Optionen überhaupt als gültig anerkannt werden. Gleichwohl bedeutet es nicht, dass Führungsrollen statisch sind, sondern sich in einem konstanten Fluss verschiedener Situationen bewegen. Entsprechend können Themen und deren Bedeutungen kontinuierlich durch verschiedene Meinungsführer:innen beeinflusst werden (Sutherland et al. 2014). Der Blick auf derart fluide Führungsebenen ist eine Ergänzung zur klassischen Sicht auf Führer:innenschaft in der Fachdebatte, die vornehmlich charismatische Führer:innen und deren spezifischen Eigenschaften in sozialen Bewegungen behandelt (Vanderslice 1988; Morris und Staggenborg 2004; Diani 2003). Im Hinblick auf die Forschungslücke zu Einflussfaktoren in Entscheidungsprozessen wird das Verständnis von Hierarchien in der Literatur jedoch wenig differenziert. Im vorliegenden Artikel geht es bei Hierarchien nicht nur um die Zuschreibung von Funktionen und Rangordnungen an Personen oder Gruppen innerhalb einer Bewegung. Vielmehr werden Hierarchien in ihrer Substanz und Dynamik als Mittel der Zuweisung von Funktion und Rang verstanden. Entsprechend bestimmen beispielsweise Wissenshierarchien, Informationsüberschüsse oder *skill sharing* (i. S. v. Teilen von Wissen) den Einfluss auf Entscheidungsprozesse. Aktivist:innen mit weniger Informationen und/oder Wissen verfügen entsprechend über weniger Einfluss.

### Basisdemokratie

Über die Rolle von Machthierarchien als Einflussfaktor hinaus beschäftigt sich ein weiterer Strang der Debatte mit der Frage, wie demokratisch soziale Bewegungen sind. In der Mehrzahl der Studien kommen die Autor:innen zu dem Schluss, dass soziale Bewegungen bemüht sind, ihren basisdemokratischen Prinzipien zu entsprechen (Maeckelbergh 2009; Della Porta 2009a, 2015, 2020; Rucht 2011; Della Porta und Rucht 2013; Kwok und Keung 2017). Freemans (1979) Primat des „Democratic Structuring“ wird dabei traditionell als Musterfolie basisdemokratischer Entscheidungsprozesse herangezogen. Demnach käme es im Idealfall nicht zu Machtmissbrauch von Eliten, da jegliche Entscheidung der Autorität der Gruppe als Ganzes unterstehe (Freeman 1979, S. 164). Vergleichsweise wenige Arbeiten diskutieren kritisch das Spannungsverhältnis zwischen demokratischen Prinzipien auf der einen und effizienter Entscheidungsprozesse auf der anderen Seite (Leach 2006, 2016; Kwok und Keung 2017; Della Porta und Felicetti 2019; Della Porta 2020). In diesem Zusammenhang illustrieren Kwok und Keung (2017) am Beispiel der Hongkonger Regenschirmbewegung, wie die Einführung direktdemokratischer Verfahren zwar die Legitimität von Entscheidungen erhöhte, die Handlungsfähigkeit jedoch bremste, da unterschiedlichen (auch radikalen) Segmenten der Bewegung unverhältnismäßig viel Raum zur Teilhabe gewährt wurde.

### Personenbezogene Merkmale

Neben den genannten Einflussfaktoren der informellen Hierarchien und der Basisdemokratie argumentieren Forscher:innen, dass personenbezogene Merkmale bzw. spezifische Expertisen ebenso eine Rolle in Entscheidungsprozessen spielen (Della Porta und Rucht 2013; Haug 2010, 2013). In ihrem Fokus auf mikropolitische Prozesse von Basisgruppen lehnt sich Bosse (2019) an Arbeiten von Nepstad und Bob (2006) an und nimmt dabei eine Unterscheidung zwischen verschiedenen Formen von Führungskapital vor, das einzelnen Mitgliedern sozialer Bewegungen zur Verfügung steht. Ähnlich wie bei Sutherland et al. (2014) ist jenes Kapital nicht statisch, sondern changierend im Zeitverlauf. Die Position innerhalb der (informellen) Hierarchie ist dabei nicht entscheidend, so dass sowohl besonders präsente als auch weniger in Augenschein tretende Mitglieder über jenes Kapital potenziell verfügen (Nepstad und Bob 2006; Bosse 2019). Darüber hinaus entfalten die Führungskapitale unterschiedliche kontextuelle Wirkung je nach Dynamiken in der jeweiligen Gruppe. Bosse (2019) identifiziert drei übergeordnete Kapitalarten, die sich den jeweils zugeschriebenen Kompetenzen und der von der Gruppe zugewiesenen Anerkennung unterteilen lassen: kulturelles, soziales und symbolisches Kapital (Bosse 2019). Unter kulturellem Kapital werden Kenntnisse über das generelle Verstehen von Belangen verschiedener gesellschaftlicher Gruppen, das (Fach‑)Wissen über das konkrete Thema (z. B. Klimawandelfolgen) und über die spezifische Situation der davon Betroffenen verstanden. Im Sinne von Bourdieu (1983) meint Soziales Kapital „die Gesamtheit (…) der Ressourcen, die mit dem Besitz eines dauerhaften Netzes von (…) Beziehungen gegenseitigen Kennens oder Anerkennens verbunden sind“ (Bourdieu 1983, S. 190). Ein besonderes Augenmerk gilt dabei der Stärke oder Schwäche dieser Beziehungen im Hinblick auf die Mobilisierung von Unterstützung im Zuge von Entscheidungsprozessen. Das Symbolische Kapital bezeichnet Erfahrungen und Ereignisse, bei denen Mitglieder einer Gruppe einen „hohen Preis für die Vertretung ihrer Ideale zahlen mussten“ (Bosse 2019, S. 51). Diese Erlebnisse würden eine nicht zu unterschätzende (Außen‑)Wirkung und entsprechende Rolle bei der Durchsetzung legitimer Deutungen spielen. Nepstad und Bob (2006) argumentieren, dass Entscheidungsprozesse in sozialen Bewegungen insbesondere von Personen mit reichem kulturellem, sozialem und symbolischem Kapital bestimmt würden. Gleichwohl weisen Van Dyke und Dixon (2013) einschränkend darauf hin, dass die Verfügbarkeit von jenem Kapital nicht automatisch mit großem Einfluss auf den Diskussionsprozess einhergeht. Vielmehr müsse ebenso die Zeit für die Akquise des Kapitals berücksichtigt werden, um beispielsweise Kompetenzen, Fachwissen oder Netzwerke auszubauen (Van den Hövel 2010; Zielińska et al. 2011; Van Dyke und Dixon 2013). Letzteres wird in jüngeren Studien hervorgehoben, die Entscheidungsstärke innerhalb sozialer Bewegungen anhand der Prominenz und Außenwirkung von Einzelpersonen in sozialen Medien messen (Tufekci 2013; Mundt et al. 2018; Leong et al. 2019).

Die Erkenntnisse aus der Organisationssoziologie, der Bewegungsforschung und der deliberativen Demokratie bilden den theoretischen Unterbau des vorliegenden Artikels. Um die Forschungslücke zu den internen Entscheidungsprozessen von FFF bearbeiten zu können, geht es vor diesem Hintergrund dabei insbesondere um die Operationalisierung der drei Dimensionen: Informelle Hierarchien (Freeman 1979, 2000; Willems und Jegers 2012); Basisdemokratie (Della Porta und Rucht 2013; Della Porta 2020) und Personenbezogene Merkmale (Nepstad und Bob 2006; Sutherland et al. 2014). Dass diese drei (und womöglich weitere) Dimensionen Überschneidungen aufweisen können, liegt in der Natur von Organisationen bzw. sozialen Bewegungen, die vom kontinuierlichen Zusammenspiel individuell-kollektiver Beziehungen getragen werden. Entsprechendes Augenmerk gilt bei der nachfolgenden Analyse der Frage von (temporaler) Relationalität: „[Leadership] is a shared activity that creates social meanings, which may be temporarily stable but always open to contestation, change and reinterpretation“ (Sutherland et al. 2014, S. 764).

## Methode

**Figure 1:**
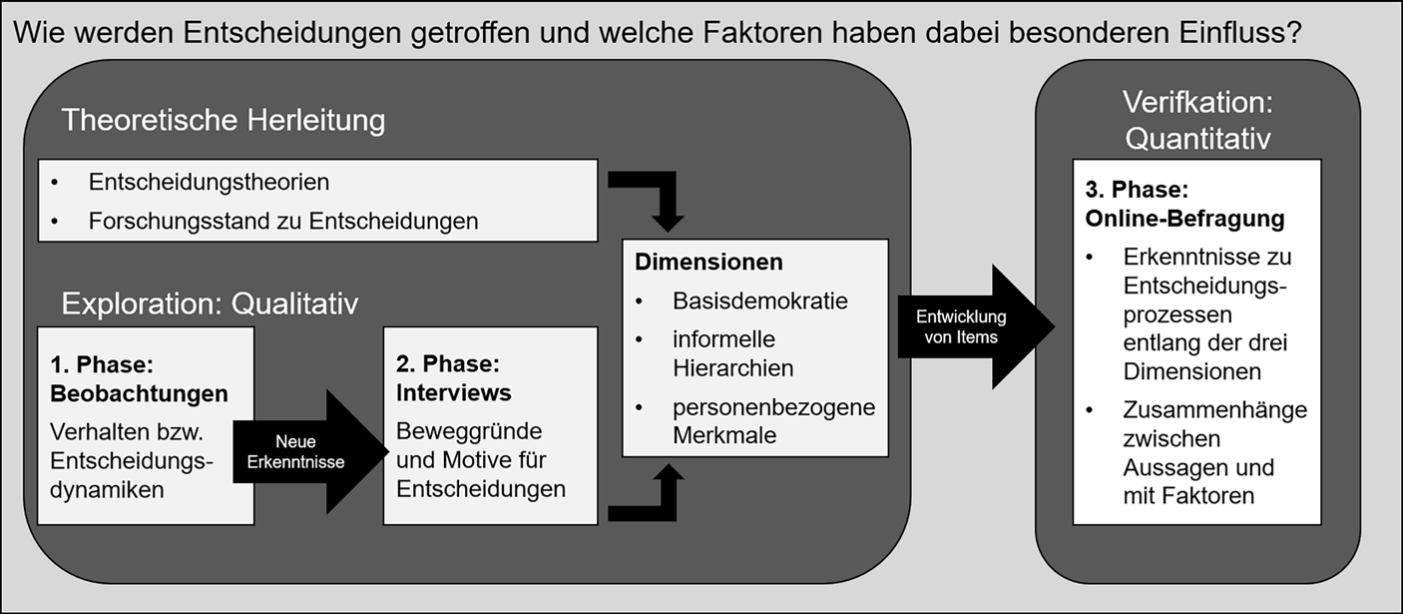

Anknüpfend an das leitende Forschungsinteresse an Entscheidungsprozessen bei FFF wurde das methodische Design auf Grundlage zweier sich ergänzender Zugänge konzipiert. Zum einen wurden die gewählten theoretischen Stränge (Basisdemokratie, informelle Hierarchien und personenbezogene Merkmale) als Basis für die Operationalisierung der Erhebung zu Grunde gelegt. Zum anderen wurde ein Mixed-Method-Design, bestehend aus teilnehmenden Beobachtungen, leitfadengestützten Interviews und einer quantitativen Online-Befragung, entwickelt (vgl. Online-Appendix). Während die theoretischen Grundlagen und der Forschungsstand eine deduktiv generierte Kategorienentwicklung ermöglichte, konnten induktive Erkenntnisse, die im Zuge der qualitativen Erhebungsphase (teilnehmende Beobachtungen und leitfadengestützte Interviews) gewonnen wurden, diese Kategorienstruktur komplementieren. Diese Ergänzungen waren richtungsweisend für den weiteren Erhebungsprozess und haben neue Impulse – beispielsweise zu den zentralen Einflussdimensionen – für Items der anschließenden Online-Befragung ermöglicht (vgl. Abb. 1). Diese Between-Method-Triangulation hat sich als fruchtbar erwiesen und ermöglichte, im Rahmen der qualitativen Erhebungsphasen explorativ über Erfahrungen der beobachteten und interviewten FFF-Aktivist:innen in die Tiefe zu forschen. Diese Erkenntnisse flossen in Form von zielgruppenspezifischen Items in die Konzeption des standardisierten Online-Fragebogens ein und konnten somit die theoretisch begründeten Annahmen zusätzlich untermauern.

Im vorliegenden Projekt wurden für die sieben teilnehmenden Beobachtungen in OGs und Delegiertenkonferenzen sowie für die zwölf Interviews mit Aktivist:innen unterschiedlicher OGs und Funktionen drei leitende Dimensionen für die jeweiligen Erhebungsphasen konzipiert, die sich letztlich um den zentralen Aspekt der Entscheidungsfindung drehten (Lamnek 2010; Helfferich 2011). Abb. 1 verdeutlicht die unterschiedlichen Vorgehensweisen und Erhebungsschritte sowie das Zusammenwirken (Pickel 2009; Lamnek 2010; Helfferich 2011; Kruse 2014; Soßdorf 2016; Ecarius und Miethe 2018; Flick 2020). So wurden die Beobachtungen vorwiegend von den Mitforschenden oder als Tandem aus Mitforschenden und etablierten Forschenden durchgeführt (Januar/Februar 2020). Hier standen vor allen Dingen die Verhaltensweisen und Gesprächsdynamiken im Vordergrund, die auf einem vorbereiteten Beobachtungsbogen stichpunktartig notiert wurden. Diese Hinweise waren für die Online-Befragungen entscheidend, da so deutlich wurde, welche der beobachtenden Abläufe, Methoden aber auch Besonderheiten sich für eine zusätzliche Fragestellung im Fragebogen eignen würden. Noch konkreter und ausführlicher konnte auf die Beweggründe und Motive solcher Entscheidungsprozesse in den Interviews eingegangen werden (Januar/Februar 2020). Hier zeichneten sich viele Überschneidungen zwischen den Interviewpartner:innen, aber auch kontroverse Erkenntnisse ab, die schließlich in eine induktive Kategorienentwicklung eingeflossen sind.

Die zentralen Ergebnisse der qualitativ durchgeführten Exploration bestätigten die theoretisch herausgearbeitete, besondere Bedeutung der drei Dimensionen (1) Basisdemokratie, (2) informelle Hierarchien und (3) personenbezogene Merkmale, die in Form weiterer, differenzierterer Items Eingang in den Fragebogen gefunden haben. So wurden Fragen rund um die eigenen Erfahrungen, aber auch Einstellungen im Zusammenhang mit Entscheidungsprozessen bei FFF formuliert. Daneben wurden Fragen konstruiert, die sich auf mögliche Einflussfaktoren bei der individuellen Einschätzung beziehen (Alter, Kompetenzen, Aktivitätsgrad), um zu eruieren, welche Zusammenhänge zwischen diesen Faktoren und den individuellen Einstellungen zu den Entscheidungsprozessen bestehen. Der Fragebogen bestand aus 22 Fragen mit Unterfragen, unterschiedlichen Itembatterien und Skalenniveaus, sodass in der Auswertung eine große Vielfalt an deskriptiven Verfahren sowie Zusammenhangsanalysen realisiert werden konnte (Porst 2011).

Hinsichtlich der Auswertungsstrategie wurde analog zur Erhebungsmethodik eine Daten-Triangulation angelegt und damit ermöglicht, dass Erkenntnisse aus verschiedenen Erhebungsphasen und aus unterschiedlichen Datenquellen miteinander verwoben werden konnten. Während die qualitativen Daten inhaltsanalytisch (Kruse 2014; Mayring und Fenzl 2019) im Hinblick auf tiefergehende, exemplarische Ergebnisse hin ausgewertet wurden, konnten diese Erkenntnisse in der quantitativen Auswertung der Online-Befragung auf breiter Basis verifiziert werden. Dabei wurden sämtliche Verteilungen der Variablen und angenommene Zusammenhänge berechnet, die zur Beantwortung der Fragestellung relevant erschienen. In der folgenden Ergebnisdarstellung und Diskussion werden allerdings nur diejenigen Zusammenhänge diskutiert, die zielführend waren für die Beschäftigung mit der übergeordneten Forschungsfrage im Hinblick auf die drei Dimensionen Basisdemokratie, informelle Hierarchien und personenbezogene Merkmale (Raithel 2008; Kuckartz et al. 2010; Diaz-Bone 2013; Flick 2020).

Die methodischen Überlegungen und die Umsetzung der unterschiedlichen empirischen Schritte fanden auf Grundlage eines Citizen-Science-Ansatzes statt (Irwin 1995; Hecker et al. 2019; Mucha et al. 2020; Haklay et al. 2021). Konkret wurden in diesem Projekt über einen Zeitraum von zwölf Monaten zehn Aktivist:innen von FFF aus dem Raum Düsseldorf in einen mehrstufigen Forschungsprozess eingebunden. In kollaborativen Workshops und mithilfe onlinegestützter Tools haben Sozialwissenschaftler:innen mit den Mitforscher:innen sukzessiv an der Entwicklung des Projektes gearbeitet. Diese widmeten sich jeweils einer anderen Forschungsphase (Forschungsfrage, Erhebungsdesign, Erhebung, Auswertung, Publikation). So erhielten die Bürgerforscher:innen einen Einblick in den sozialwissenschaftlichen Forschungsprozess und konnten zugleich neue Ideen und Impulse für die jeweils anstehenden Projektphasen einbringen. Da die Mitforscher:innen außerhalb des akademischen Betriebs verortet waren und gleichzeitig über ein breites Wissen in Bezug auf FFF verfügten, haben sich im Verlauf ungeahnte, kreative und innovative Ideen für alle Phasen ergeben. Ein nicht zu unterschätzender Mehrwert der Zusammenarbeit mit den Aktivist:innen war letztlich der Zugang zu den Ortsgruppensitzungen, den Interviewpartner:innen und den Netzwerkstrukturen für die Streuung der Online-Umfrage.

Eine besondere Herausforderung dieses Ansatzes ist der Umgang mit wissenschafts-ethischen Aspekten. Aus diesem Grund wurde zu Beginn der Zusammenarbeit mit den Mitforschenden im Rahmen des ersten gemeinsamen Workshops über wissenschaftliche Gütekriterien gesprochen und deutlich gemacht, wie objektive Ergebnisse generiert und reliable Erkenntnisse in der Wissenschaft gewonnen werden können. In diesem Zuge wurde vermittelt, dass die Mitforschenden zu jeder Zeit des Projektes entscheiden können, ob ihre Mitarbeit in Publikationen und Vorträgen namentlich erwähnt werden soll. Ebenso war ihnen freigestellt, ob sie bei den internen virtuell gehaltenen Workshops mit Namensnennung und Bild teilnehmen möchten. Eine entsprechende Einwilligungserklärung der Teilnehmenden zur Mitwirkung am Projekt sowie zum Datenschutz wurde im Vorfeld unterzeichnet; bei Minderjährigen wurde diese über die Eltern realisiert. Insgesamt erfolgte die Zusammenarbeit zwischen den Mitforschenden und etablierten Forschenden in allen Phasen des Projektes auf Augenhöhe. Sie waren zu jeder Zeit in alle Forschungsschritte involviert. Jeder der beteiligten Personen wurde eine Stimme gegeben, so dass die besseren Argumente und nicht Hierarchien über die Ausgestaltung und den Fortgang des Vorhabens bestimmten. Entsprechend wurden Entscheidungen zum Forschungsgegenstand, der Methode und der Interpretation nach Konsens- und nicht nach Mehrheitsprinzip gefällt. Bezeichnenderweise kam der Impuls, sich mit internen Entscheidungsprozessen zu beschäftigen, von Seiten der Mitforschenden. Gleichwohl muss in der Natur der Sache einschränkend festgehalten werden, dass, aufgrund ihrer fehlenden wissenschaftlichen Erfahrungen und parallelen Verpflichtungen (z. B. Abiturvorbereitungen, Klausurphasen), die Mitforschenden weniger Zeit in das Projekt investieren konnten als die Forscher:innen. Angesichts der bewussten und vorgelebten flachen Hierarchien entwickelte sich ein Vertrauensverhältnis, das es ermöglichte, auch (selbst-)kritische Problematiken offen anzusprechen und in der Gruppe gemeinsam zu reflektieren. So hinterfragten beispielsweise die Mitforschenden im Vorfeld der teilnehmenden Beobachtungen ihre Doppelrolle als Aktivist:in und Forscher:in zugleich gegenüber der eigenen Bewegung. Der besondere Vorteil des Citizen-Science-Ansatzes war in solchen Zusammenhängen, dass zum einen gemeinsam ein sensibilisiertes Verständnis für die sozialwissenschaftliche Intervention erarbeitet wurde (z. B. Schulung zum Verhalten während der Beobachtung, Entwicklung eines Kriterienkatalogs der zu beobachtenden Interaktionen). Zum anderen floss die Thematisierung jenes Loyalitätskonflikts in das wissenschaftsethische Framework des Vorhabens ein. Bereits vor Beginn der Zusammenarbeit mit den jungen Aktivist:innen hatte sich das Team der etablierten Forscher:innen im Sinne des Citizen-Science-Ansatzes darauf geeinigt, dass nur ein hoher wissenschaftsethischer Anspruch an die Bedeutung der Mitforschenden und ihrer Souveränität in erster Linie als Bürger:in mehr Beachtung geschenkt werden müsse als den empirischen Ergebnissen. Diese Grundsatzentscheidung förderte die Vertrauensbasis und ermöglichte letztlich die Durchführung des Projekts.

Zum Zeitpunkt der Auswahl der anzuschreibenden OGs im April 2021 hatte FFF auf ihrer offiziellen Website deutschlandweit 676 Gruppen aufgeführt (Fridays for Future Germany 2021a). Auf dieser Liste standen nicht nur Messenger-Kanäle einzelner Orte, sondern auch regionale oder überregionale Gruppen von FFF. Von diesen Gruppen wurden zufällig 500 ausgewählt. Den zufällig gezogenen Gruppen wurde über die Messenger-App Whatsapp beigetreten; wenn dieser Kontaktkanal nicht zugänglich war, alternativ über Telegram. In einigen Fällen war der Beitritt zu den Gruppen über beide Wege nicht möglich, daher fiel die Kontaktaufnahme bei 58 Gruppen aus. Somit konnte die Umfrage final an 442 (Orts‑) Gruppen über Whatsapp oder Telegram geschickt werden. Zusätzlich haben die Mitforscher:innen des Citizen-Science-Projekts die Umfrage intern verbreitet. Schlussendlich haben 784 Personen die Umfrage gestartet und sie wurde von 507 Teilnehmer:innen beendet. Somit lag die Beendigungsquote bei 65 %. Der Erhebungszeitraum lief vom 16.04.2021 bis 23.05.2021. Auch im Hinblick auf die durchgeführten Interviews, die Beobachtungen und die Online-Umfrage wurde auf die Einhaltung der Datenschutzbestimmungen und die Zusicherung der vollständigen Anonymisierung geachtet. Dazu wurden alle Daten auf einen datenschutzkonformen Cloudserver mit Sitz in Deutschland und besonderen Anforderungen für Universitäten (sciebo) gelegt und auch die Plattform für die Online-Umfrage wurde über den eigenen lizenzierten Zugang der Universität Düsseldorf umgesetzt.

## Ergebnisse

Mit Blick auf die eingangs formulierte Fragestellung nach der Art und Weise, wie Entscheidungen bei FFF getroffen werden und welche Rolle spezifische Einflussfaktoren spielen, ermöglichte die Kombination der qualitativ und quantitativ erhobenen Daten zweierlei: Einerseits konnten tiefgehende Einschätzungen zu komplexen Zusammenhängen, Motiven und Hindernissen im Hinblick auf die inneren Strukturen bei FFF aufgedeckt werden. Andererseits konnte in der Breite aufgezeigt werden, wie weit verstreute Aktivist:innen in Deutschland der Bewegung gegenüber eingestellt sind. Im Folgenden werden die zentralen Erkenntnisse zunächst hinsichtlich allgemeiner Aussagen zur Entscheidungsfindung und anschließend entlang der drei Dimensionen Basisdemokratie, informelle Hierarchien und personenbezogene Merkmale präsentiert.

Die nähere Betrachtung der tatsächlichen Prozesse innerhalb der Bewegung steht im Fokus des hiesigen Erkenntnisinteresses und bildet den Schwerpunkt der folgenden Ergebnisdarstellung. Vorab gilt festzuhalten, dass die Ergebnisse zu den soziodemografischen Merkmalen der Befragten im Wesentlichen mit bisherigen Untersuchungen übereinstimmen und die Unterschiede des aktiven Kerns der Bewegung im Vergleich zu den Teilnehmer:innen an Demonstrationen unterstreichen (De Moor et al. 2020; Mucha et al. 2020). Von den 495 Personen, die eine Angabe zu ihrem Alter machten, waren die größten Altersgruppen die 16- bis 17-Jährigen (30,3 %) und 18- bis 19-Jährigen (29,1 %). Über 22-Jährige sind nur mit knapp 11 % vertreten. Insgesamt stützt die demografische Verteilung die Werte früherer Befragungen der in OGs aktiven Aktivist:innen (Mucha et al. 2020) und unterstreicht die Unterschiede des aktiven Kerns der Bewegung im Vergleich zu Teilnehmer:innen an Demonstrationen. Ähnlich lautet der Befund für die Verteilung der Geschlechter in der Gruppe der Befragten. Weibliche Personen stellen die klare Mehrheit mit 57,4 %. Etwa 37 % identifizieren sich als männlich, 5,5 % als divers. Auch diese Zahlen reflektieren die Verteilung bei Mucha et al. (2020) und De Moor et al. (2020). Von den Befragten sind 56 % mindestens ein Jahr aktiv bei FFF und weitere 22 % ein halbes Jahr. Hinsichtlich der Engagementintensität wird deutlich, dass 65 % der Aktivist:innen regelmäßig an Treffen von FFF teilnehmen, 15 % in einer AG auf Bundesebene aktiv sind und 38 % als Delegierte ihrer OG gewählt wurden. Die meisten Befragten (42 %) kommen aus mittelgroßen Städten (20.001 bis 100.000 Einw.), gefolgt von Großstädten (24 %; 100.001 bis 500.000 Einw.) und Kleinstädten (20 %; 5001 bis 20.000 Einw.).

### Entscheidungsprozesse

Die Entscheidungen in den OGs sowie den zahlreichen AGs auf der Orts- und Bundesebene bei FFF erfolgen formal stets nach dem gleichen basisdemokratischen Grundprinzip: Alle Mitglieder sind stimmberechtigt und können frei an den Abstimmungen teilnehmen. Alle Interessierten können an FFF-Ortsgruppentreffen teilnehmen. Dabei gibt es keine Mitgliedschaft im offiziellen Sinne sowie keinen formalisierten Vorgang oder bestimmte Voraussetzungen, die zu erfüllen sind. Formale Grundlage für Entscheidungsfindungen ist ein eigenes Strukturpapier (Fridays for Future 2021b), in welchem die Abstimmungsmodalitäten festgelegt sind und welches für die gesamte Bewegung gilt. Dieses Mehrheitsprinzip, nach dem die Entscheidungen in aller Regel gefällt werden, wird in den Interviews mit den FFF-Aktivist:innen bestätigt: „Abstimmungen sind gleich, egal ob OG oder AG, jede:r hat eine Stimme, alle dürfen abstimmen, wir entscheiden dann nach Mehrheit“ (Interview 1). In seltenen Ausnahmefällen, wenn etwa das Stimmungsbild nicht eindeutig ist, kommt in vereinzelten OGs ein mehrstufiges Konsensverfahren zum Einsatz, um eventuelle Bedenken einzelner Aktivist:innen aufzuheben (vgl. Interview 2). Die OGs sowie die dazugehörigen AGs bilden das demokratische Herzstück von FFF. Sie können unabhängig von der Bundesebene agieren und dennoch maßgeblich bundesweite Entscheidungen beeinflussen. Abstimmungen finden in den regelmäßig stattfindenden Sitzungen statt, die entlang einzelner Tagesordnungspunkte (TOP) gegliedert sind. Diese TOPs wiederum können von allen Mitgliedern vorab eingebracht werden und sind häufig mit zusätzlichen Hinweisen und Argumenten versehen, um eine transparente Informationslage sowie Orientierungshilfen für die Abstimmenden zu gewährleisten. Bei der Entscheidungsfindung berücksichtigen die OGs zahlreiche Aspekte, wie beispielsweise die zur Verfügung stehenden Kapazitäten (vgl. Interview 3). Solche Prozesse können mitunter mehrere Wochen dauern (vgl. ebd.) „[…] in der Tagesordnung werden schon Pro- und Contra-Argumente gesammelt und da ist das schon sehr strukturiert. Wir haben auch festgelegt, wann was kommt. Also zuerst kommen in der TO Berichte und am Ende kommen die Diskussionen, damit man quasi ein Konzept hat […]“ (Interview 3).

Zur Herbeiführung von Entscheidungen mit bundesweiter Tragweite wählt jede OG Stellvertretende, die in ihrer Funktion als Delegierte die Schnittstelle zwischen OG und Bundesebene bilden. Im Gegensatz zu den regulären öffentlichen Abstimmungen finden Personalwahlen anonymisiert statt (vgl. Interview 4). Im Rahmen von Telefonkonferenzen – den sogenannten Deli-TK’s – tauschen sich die Delegierten aus. Gemäß des Strukturpapiers (Fridays for Future 2021b, S. 8) hat jede OG eine Stimme, wodurch alle partizipierenden Gruppen über das gleiche Stimmgewicht verfügen. Die Entscheidungsprozesse auf der Bundesebene weisen jedoch einige Besonderheiten auf. So müssen Anträge mit Angaben zum Abstimmungsinhalt und den Entscheidungsmöglichkeiten vorab an eine eigens dafür eingerichtete Communications Task Force (CTF) übermittelt werden (vgl. Interview 5). Weiterhin besteht für jede OG die Möglichkeit, ein Veto einzulegen (vgl. Interview 1). Diese Option unterbricht vorübergehend die Abstimmung und setzt einen Kompromissfindungsprozess in Gang, in dem sich weitere OGs dem Veto anschließen und per Telefonkonferenz diskutieren können. Dieser Vorgang wird moderiert und endet im positiven Fall mit der Aushandlung eines Kompromisses und einer erneuten Abstimmung (vgl. Interview 1). „Aber wenn es wirklich so richtig kontroverse Anträge gibt, dann legen OGs schonmal Vetos ein, damit ist die Abstimmung dann ab dem Moment unterbrochen. Und es muss ein Kompromissfindungsprozess starten“ (Interview 5).

Werden die Zusammenhänge der Online-Befragung zu der Zufriedenheit mit den Abläufen, den Diskussionen und der Einbindung der unterschiedlichen Meinungen betrachtet, so wird deutlich, dass es große Teile der Bewegung gibt, die diese Ansprüche erfüllt sehen. So gibt es eine hohe Zustimmung zu den Items „Zufriedenheit mit Diskussionen“ und „Meine Meinung wird ausreichend berücksichtigt“ (mit Mittelwerten von 5,2 bzw. 6 auf einer Skala von 1–7 und einer starken Pearson-Korrelation von r = 0,54***). Sowohl in den Beobachtungen als auch den Interviews wurde allerdings deutlich, dass der Anspruch des Mehrheitsprinzips in der Praxis häufig herausgefordert wird, da Diskussionen unter Zeitdruck teilweise abgekürzt werden, Meinungen einer Minderheit schneller abgewiesen werden und einzelne moderierende Akteur:innen – stärker als in ihrer Rolle vorgesehen – Einfluss auf die Entscheidungsfindung nehmen (vgl. Beobachtung OG Großstadt, Deli-TK online). So werden im eigentlichen Prozess die Rollen unter Umständen eher schwammig ausgelegt:

„In diesem Strukturpapier steht eigentlich, dass die AG-Sprechenden – halt so wie Klassensprechende – eigentlich keine Entscheidungsgewalt haben, sondern nur repräsentieren. […] Es ist auch häufig immer noch so, dass dann informell die AG-Sprechenden dann schon eine Entscheidungsgewalt haben, die aber nirgendwo festgeschrieben ist“ (Interview 6).

### Basisdemokratie

**Figure 2:**
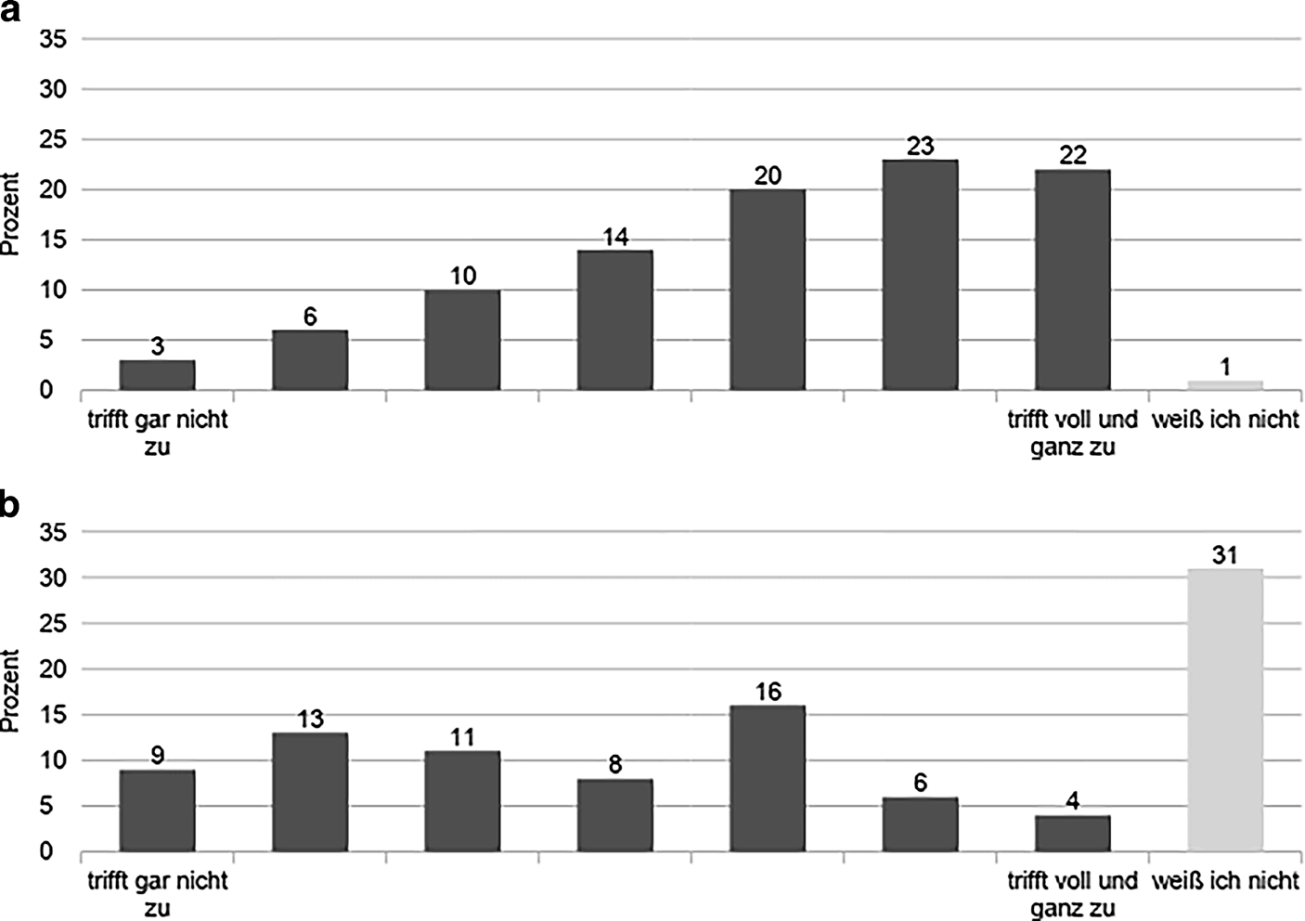

Der basisdemokratische Ansatz wird grundsätzlich von einer Mehrheit der Befragten getragen. Etwa zwei Drittel der Aktivist:innen stimmte der Aussage zu „Es sollten immer alle Aktivist:innen an einer Entscheidung beteiligt werden“. Bei dieser und den folgenden Aussagen wurden jeweils auf einer Skala von 1–7 die Werte 5–7 als Zustimmung und 1–3 als Ablehnung gewertet. Es zeigt sich jedoch eine Diskrepanz zwischen dem Ideal und dem gelebten Aktivismus. 26 % der Aktivist:innen gaben an, dass die tatsächlich gelebten Abläufe bei Entscheidungen von dem basisdemokratischen Selbstverständnis der Bewegung abweichen (Abb. 2). Nur etwa ein Drittel gab an, dass das eher nicht oder gar nicht zuträfe. Dabei zeigten sich kaum Unterschiede zwischen Delegierten und Nicht-Delegierten bei der Frage, ob die gelebten Abläufe von dem Selbstverständnis abweichen. Einschränkend muss ergänzt werden, dass ein weiteres Drittel der Befragten „weiß nicht“ angab. Die Ausweichkategorie benutzten dabei Personen ohne Erfahrung auf Bundesebene deutlich häufiger als diejenigen, die auf der Bundesebene engagiert waren oder sind (62 % vs. 38 %). Dies ließe sich unter anderem dadurch erklären, dass sich Aktivist:innen, die nur auf der Ortsebene aktiv sind, eine Einschätzung nicht zutrauten.

**Figure 3:**
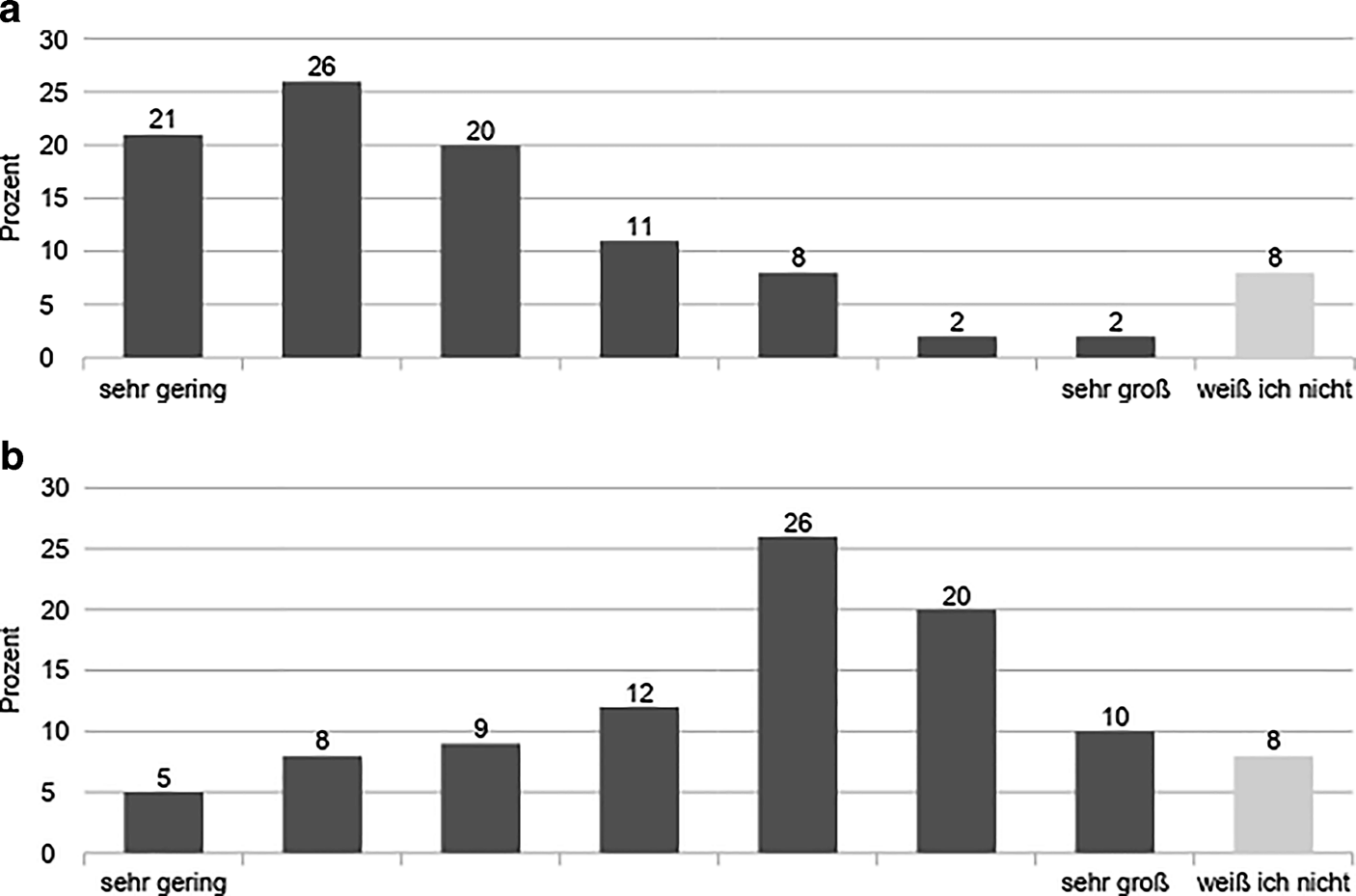

Auffällig ist, dass die Einschätzung des eigenen Einflusses stark unterschiedlich ist, je nachdem ob er auf die gesamte Bewegung oder nur die eigenen Gruppen bezogen wurde. Nur 12 % gaben an, dass ihr Einfluss auf die Bewegung groß sei, wohingegen 56 % einen großen Einfluss auf die Gruppen der Bewegung, in denen sie aktiv seien, angaben (Abb. 3). Diese Zahlen zeigen einerseits, dass der basisdemokratische Anspruch teilweise dort erfüllt wird, wo Aktivist:innen sich unmittelbar engagieren. Dies bezieht sich jedoch andererseits nicht auf die Bewegung insgesamt, bei der etwa zwei Drittel angaben, nur geringen Einfluss zu besitzen (Abb. 3).

Dass nicht alle Personen gleichen Einfluss auf Entscheidungen haben, wie es der basisdemokratischen Vorstellung Freemans (1979) entsprechen würde, wird auch in der Selbsteinschätzung der Befragten reflektiert. Von den Delegierten, die eine Angabe machten, sagten 70 %, dass ihr Einfluss in den Gruppen der Bewegung, in denen sie aktiv seien, eher groß bis sehr groß sei. Unter Nicht-Delegierten gaben dies nur 55,5 % an. Hier zeigt sich ein klares Gefälle in dem Einfluss, den Personen der Bundesebene auf ihre Gruppen haben im Vergleich zu den Personen, die nicht auf Bundesebene aktiv sind oder waren. Eine auf Bundesebene aktive Person erklärt diesen Umstand mit dem Aufwand, der mit der Konsultation anderer Aktivist:innen verbunden ist:

„Aus dem Grund, dass ich nicht genug Zeit habe […] und tendenziell wählen muss zwischen dem Einholen weiterer Meinungen, die mich oder das Thema weiterbringen, dann führe ich das lieber allein durch, auch ohne Basisdemokratie. Wie gesagt, geht es mir dabei um die Arbeitsfähigkeit“ (Interview 7).

Wie in der Folge diskutiert wird, macht diese Analyse deutlich, dass die untersuchten Dimensionen eng miteinander zusammenhängen und sich durchaus überschneiden (Sutherland et al. 2014). Im folgenden Abschnitt werden informelle Hierarchien analysiert, die trotz des basisdemokratischen Grundsatzes entstanden sind.

### Informelle Hierarchien

**Figure 4:**
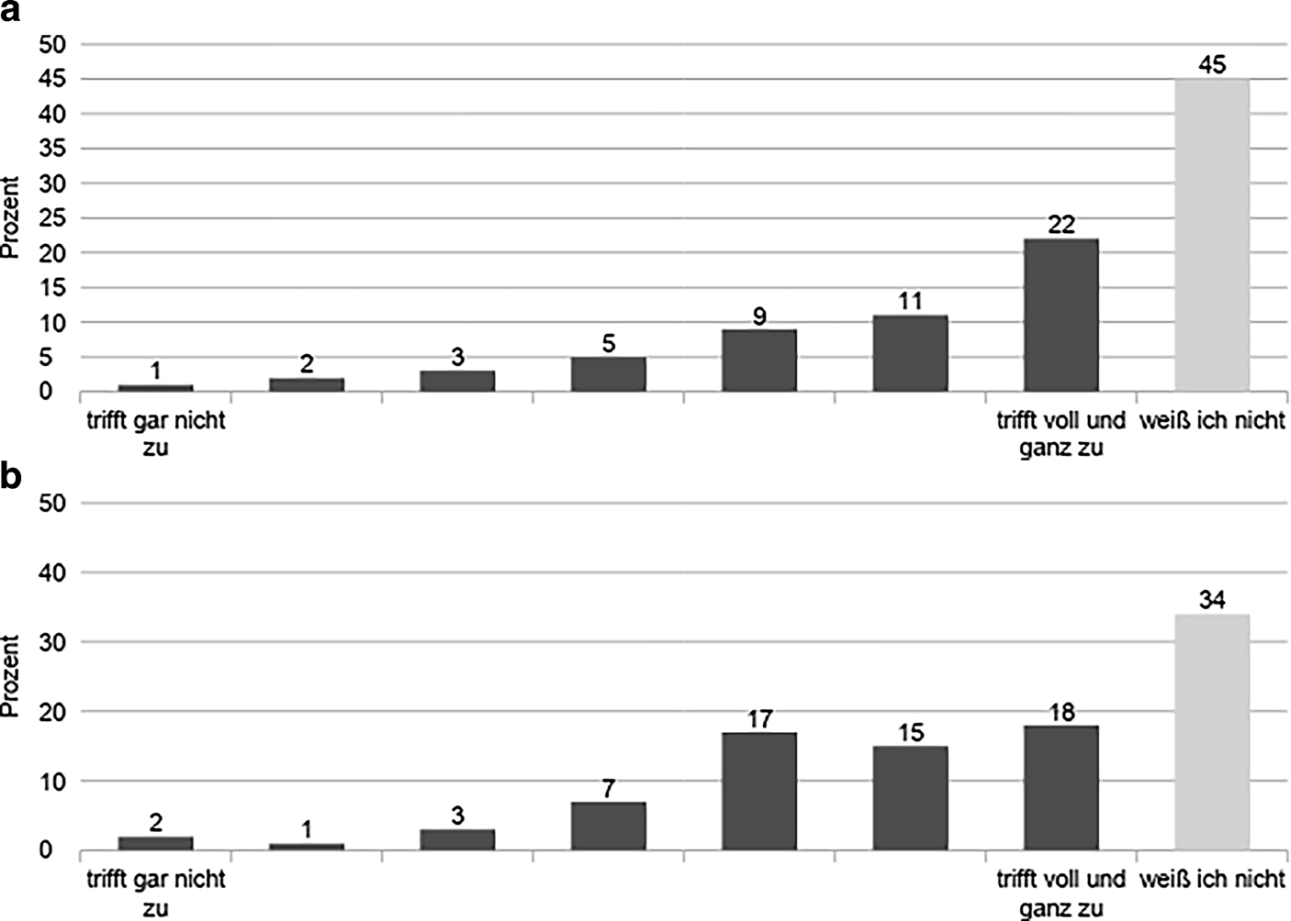

Wegen der basisdemokratischen Grundhaltung von FFF gibt es in formeller Hinsicht keine hierarchische Gliederung der Bewegung. Auffällig ist jedoch auch hier erneut die hohe Anzahl an Non-Responses bei ausgewählten Items. So hat fast die Hälfte der befragten Aktivist:innen die Frage nach der Existenz von informellen Hierarchien auf der Bundesebene nicht beantwortet (Abb. 4). Dabei zeichnen sich Unterschiede zwischen den Mitgliedern ab. Das Antwortfeld „weiß ich nicht“ haben Personen ohne Delegierten-Erfahrung mit 57 % knapp doppelt so häufig gewählt wie aktive und ehemalige Delegierte, welche diese Antwortoption zu jeweils ca. 30 % auswählten. Die übrige Antwortverteilung lässt allerdings eine starke Tendenz zur Bestätigung dieser Feststellung erkennen. Demnach trifft die darin abgefragte Aussage, dass es auf der Bundesebene informelle Hierarchien gibt, laut 42 % aller Befragten zu bzw. voll und ganz zu, wohingegen lediglich 6 % diese Aussage negierten. Bei genauerer Betrachtung zeichnen sich auch hier Unterschiede zwischen den Mitgliedern ab, da 57 % der aktiven und ehemaligen Delegierten dieser Aussage zustimmten und 33 % der Personen ohne Erfahrungen auf der Delegiertenebene.

Das Zustandekommen von informellen Hierarchien lässt sich insbesondere auf ein Informationsungleichgewicht innerhalb der Bewegung zurückführen. So kommt es zu Wissenshierarchien zwischen den verschiedenen Ebenen, die von einigen Mitgliedern als Informationsasymmetrie wahrgenommen werden: „Wir haben natürlich Wissenshierarchien, weil wir Leute haben, die viel Erfahrung haben und Menschen, die fast keine Erfahrung haben“ (Interview 3). Die Aggregation von Wissen und Erfahrungen einzelner Mitglieder sowie dessen Monopolisierungspotential können somit zur Entstehung und Manifestation informeller Hierarchien beitragen, sowohl intentional als auch aus der Dynamik des Prozesses heraus. Wenn es um informelle Hierarchien geht, spielen auch Freundeskreise und Netzwerke eine große Rolle, weil darin Wissen geteilt und Informationen ausgetauscht werden. Solche Kontakte können beispielsweise in den AGs geknüpft werden oder in den Telefonkonferenzen auf Delegiertenebene ausgebaut werden. Dies kann wiederum zu Verzerrungen bei Diskussionen und Entscheidungsprozessen führen, weil sich gut vernetzte Mitglieder im Vorfeld von Entscheidungen besprechen und gemeinsame Positionen entwickeln können.

„Meine Erfahrung der letzten zwei Jahre ist, […] dass es manchmal nicht so gut funktioniert, wenn man alle Leute bei allen Punkten einbindet. Manchmal ist es bequemer, es nicht zu tun bzw. in den jeweiligen Gruppen zuerst zu besprechen. Manchmal ist es entspannter, wenn nicht alles ausdiskutiert werden muss“ (Interview 7).

In diesem Zusammenhang gibt es innerhalb der Bewegung eine Kontroverse darüber, inwiefern es eine sogenannte *Bundesorga* gibt. Damit ist ein bestimmter Personenkreis gemeint, der an der Spitze von FFF steht und die Ausrichtung der Bewegung faktisch bestimmt. „Es gibt natürlich auf Bundesebene schon relativ enge Freundeskreise teilweise. So ganz konkret haben wir, glaube ich zwei, drei, die wirklich eng auch zusammenarbeiten“ (Interview 5). Der Existenz eines solchen Zirkels stimmten 50 % aller Befragten zu, während dies laut 6 % nicht zutrifft. Wie bereits im oben genannten Item bezüglich informeller Hierarchien ist die Antwortrate zur Option „weiß ich nicht“ mit 34 % sehr hoch (Abb. 4). Bei dieser Ausweichkategorie zeigt sich eine Diskrepanz zwischen Personen ohne Erfahrung auf der Delegiertenebene (41,5 %) und aktiven (24,9 %) bzw. ehemaligen Delegierten (22,5 %).

### Personenbezogene Merkmale

Ähnlich wie die Haltung der Befragten zur Basisdemokratie und den informellen Hierarchien liefern auch die Einschätzungen zu persönlichen Faktoren einige aussagekräftige Befunde. So wurde in fast allen Interviews deutlich, dass bestimmte Kompetenzen, Eigenschaften und Erfahrungen häufig einen Vorteil haben können, wenn es um den eigenen Einfluss auf bestimmte Entscheidungsprozesse geht: „Natürlich haben z. B. Luisa [Neubauer], Carla [Reemtsma] und so […] immer noch mehr Einfluss auf Entscheidungen. Das ist wahrscheinlich, weil sie deutlich mehr Erfahrung haben als die meisten. Weil sie wirklich sehr kompetent sind“ (Interview 8). Des Weiteren wurde geäußert, dass durchaus auch die Fähigkeit, sich gut vernetzen zu können, aber auch die Dauer oder die Intensität des Engagements bei FFF einen hohen Einfluss darauf haben, wie stark Entscheidungen beeinflusst werden können:

„Dann gibt es aber z. B. auch Leute, die einfach unheimlich viel bei FFF machen […] dadurch halt ein Know-How haben und einen Überblick über die Dinge, […] und die dadurch irgendwie vielleicht bei Entscheidungen irgendwie manchmal mehr die Entscheidung prägen als andere Leute“ (Interview 11).

**Figure 5:**
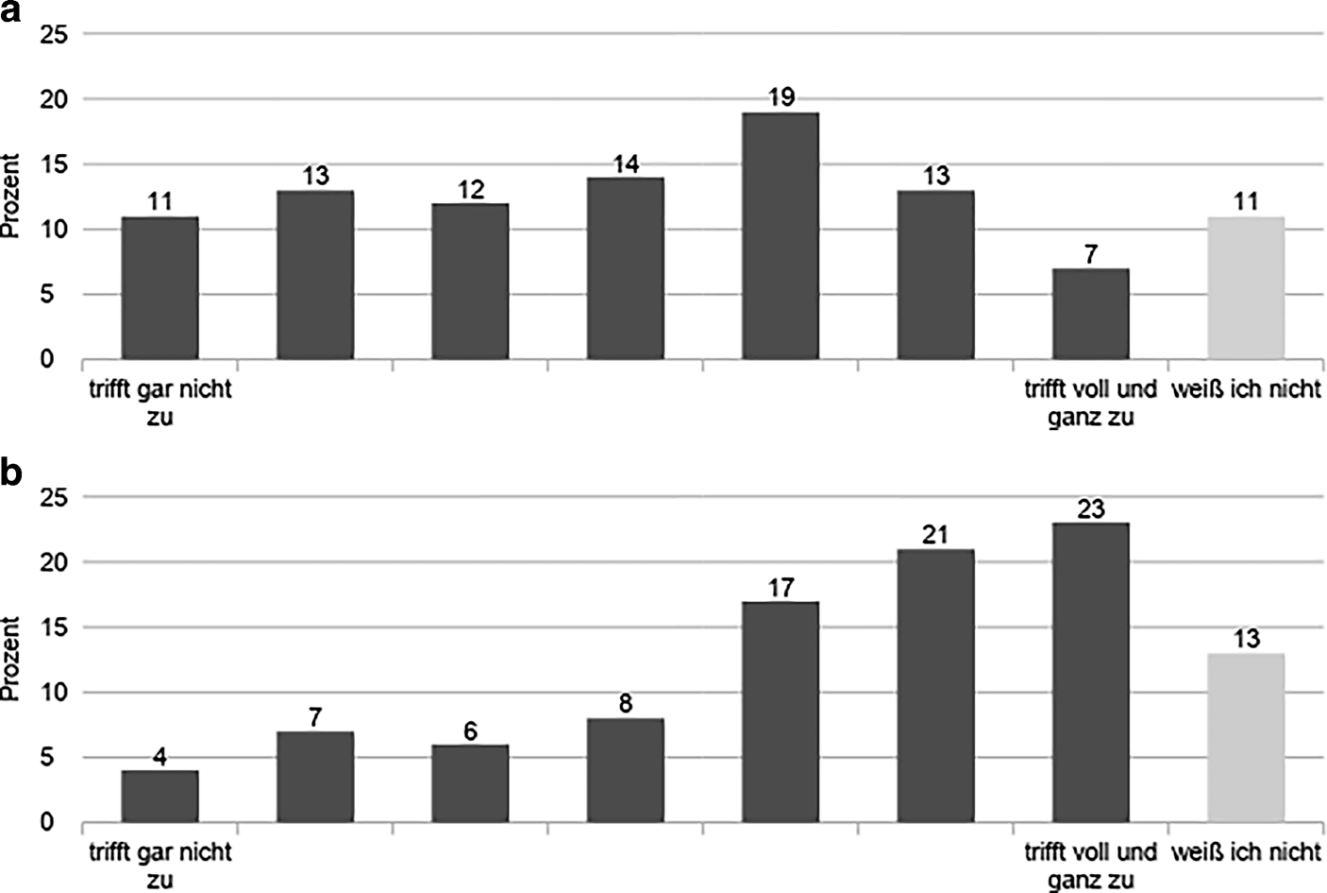

In der Auswertung der Online-Befragungen konnten nicht alle dieser Äußerungen auf einer breiten Basis verifiziert werden. So gab es klare Bestätigungen bezüglich der Zusammenhänge mit hohen Kompetenzen, der Engagementdauer und den Kommunikationsskills. Bezüglich des Netzwerkens wurde allerdings in besonderer Weise deutlich, dass die Vernetzung innerhalb der Bewegung wesentlich wichtiger eingestuft wurde für die Wirkkraft bei Entscheidungsprozessen als gute Kontakte zu Organisationen und Akteur:innen außerhalb der Bewegung. So wird in Abb. 5 sichtbar, dass die Verteilung der Zustimmungen zu der Aussage, ob Aktivist:innen bei FFF mehr Einfluss haben, wenn diese in Parteien, NGOs oder Bürger:inneninitiativen vernetzt sind, recht heterogen ist. Allerdings ist im Vergleich dazu die Bedeutung einer Vernetzung innerhalb von FFF wesentlich relevanter für eine Einflussnahme auf die Bewegung. So bestätigen dies als mindestens „eher zutreffend“ 61 % der Befragten (Abb. 5).

Diese Beobachtung wurde bereits in den Interviews deutlich: „Je besser man Leute innerhalb der Bewegung kennt, die vielleicht auch an anderen Bereichen arbeiten, die aber korrelieren, desto besser kann man Sachen vernünftig vorantreiben“ (Interview 9).

**Figure 6:**
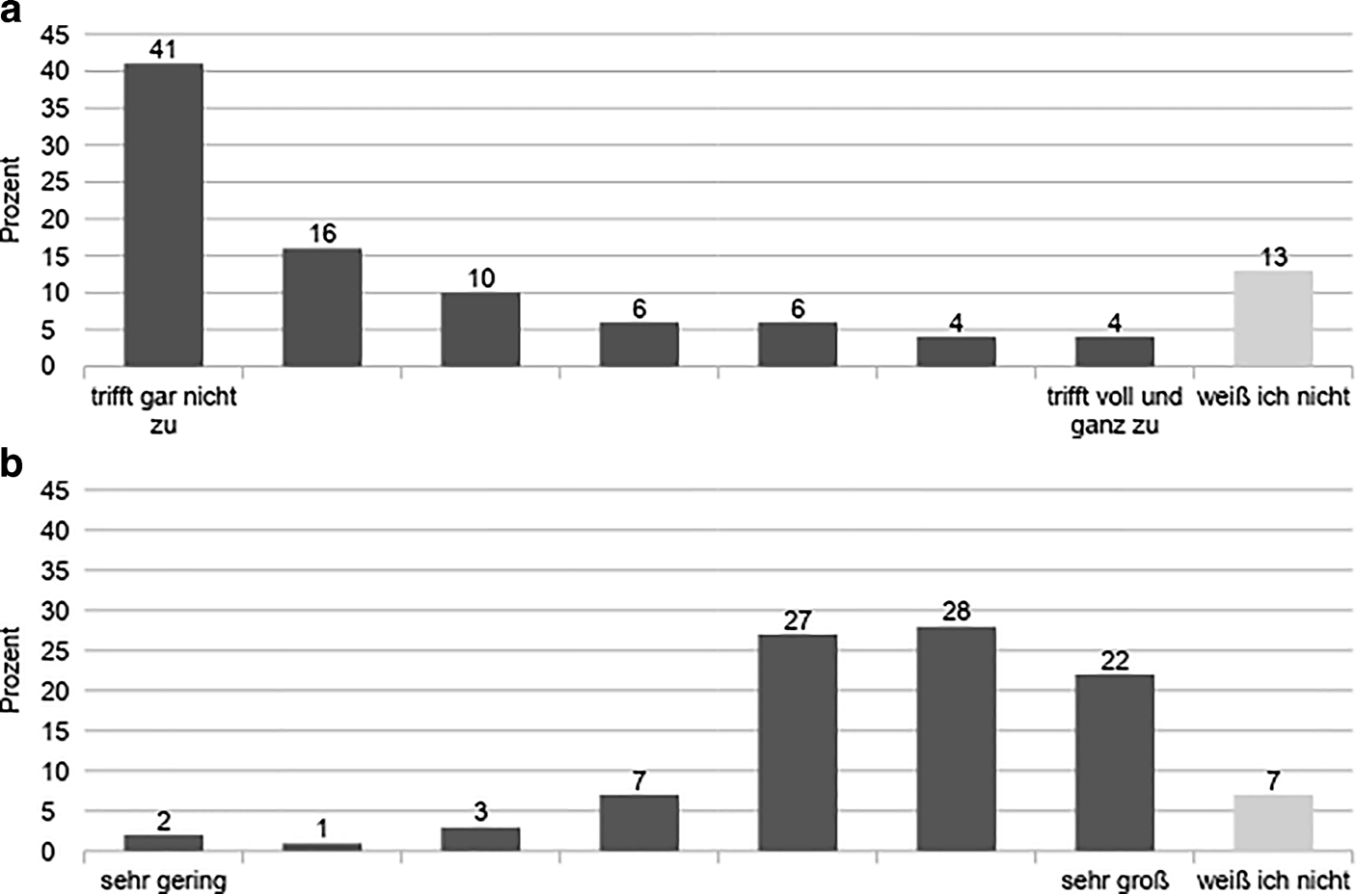

Gleichzeitig scheint sich die Bedeutung der Popularität in zwei Lager zu spalten. Demnach ist es durchaus dienlich für den Einfluss auf Entscheidungen, wenn Aktivist:innen in den klassischen Medien im Vordergrund stehen (77 % als mindestens „eher großer“ Einfluss), als dass sie viele Follower:innen in den sozialen Medien haben (67 % mindestens „eher unzutreffend“, Abb. 6). An dieser Stelle muss eingeräumt werden, dass die beiden Fragen mit unterschiedlichen Skalierungen eingeführt wurden („trifft gar nicht zu“ vs. „sehr gering“; „trifft voll und ganz zu“ vs. „sehr groß“). Für die bessere Verständlichkeit der beiden Fragen war das zuträglich. Im Hinblick auf die Auswertung ist allerdings lediglich eine eingeschränkte Vergleichbarkeit möglich. Dennoch sind die Skalen inhaltlich konsistent aufgebaut, da beide Pole eine Positionierung zwischen einer niedrigen und einer hohen Zustimmung zur jeweiligen Einflussnahme avisieren (Abb. 6).

Ein derart eindeutiger Zusammenhang hat sich in den Interviews nicht abgezeichnet. Vielmehr wurde auf ein Zusammenspiel der Inhalte in den klassischen Medien und auf den populären Social Media Profilen hingewiesen: „Aber Luisa [Neubauer] ist auf Twitter größer als der gesamte FFF-Kanal. Die postet das auf Twitter, die Medien nehmen das als Sprecherin von FFF auf und dann sind wir gezwungen, um Einigkeit zu bewahren, dieses Thema tatsächlich genau so abzudecken, oder wir riskieren den Konflikt“ (Interview 10). Gleichzeitig treten Aspekte, die als mögliche einflussreiche Faktoren erwartet wurden, in den Hintergrund, da andere Kompetenzen als bedeutsamer eingestuft wurden. So wurde im Hinblick auf die Augenhöhe zwischen Aktivist:innen erläutert, dass viel mehr Wert auf bestimmte Erfahrungen und Kompetenzen gelegt wird als auf große Altersunterschiede (Interview 7).

Im Zuge der Interpretation der Ergebnisse aus den unterschiedlichen Datenquellen wurde deutlich, dass sich die Erkenntnisse grundsätzlich ergänzen und bestätigen. Die qualitativ und quantitativ erhobenen Aussagen leuchten relevante Facetten der Entscheidungsprozesse von FFF aus und bereichern den Diskurs um tiefergehende Einblicke in das Innenleben der Bewegung.

## Fazit

Die Analyse der Beobachtungen, der Interviews und der Online-Umfrage zeigt, dass die Entscheidungsprozesse bei FFF grundsätzlich mit den selbst gesetzten Vorgaben im Strukturpapier korrespondieren und dabei insbesondere von drei Faktoren bestimmt werden. Erstens wird deutlich, dass Entscheidungen auf Bundesebene offenbar weniger basisdemokratisch getroffen werden als auf Ortsgruppenebene. Allerdings zeugt dies weniger von Machtgefällen zwischen einer elitären *Bundesorga* und einer vermeintlich ungehörten Masse auf lokaler Basis. Eine deutschlandweit operierende und dezentral organisierte Bewegung wie FFF bedarf schlichtweg arbeitsteiliger Mechanismen, um handlungs- und entscheidungsfähig zu bleiben. In einem ähnlichen Zusammenhang lässt sich zweitens feststellen, dass informelle Hierarchien auf Bundesebene eher von aktuellen und ehemaligen Delegierten wahrgenommen werden als von Nicht-Delegierten. Neben dem Einfluss von Netzwerken und Freundeskreisen werden informelle Hierarchien insbesondere auch von Informationsungleichgewichten bestimmt. Lange Mitgliedschaft und Erfahrungen innerhalb von FFF sowie spezifisch relevante Kompetenzen (z. B. Öffentlichkeitsarbeit, Kampagnenarbeit) gehen einher mit einem größeren Wirkungsradius bei Entscheidungsprozessen. Gleichwohl sind die jeweiligen Funktionen und Rollen innerhalb der internen Hierarchien im Lichte von Sutherland et al. (2014) durchaus fluide. Relevante journalistische Fertigkeiten von neuen Mitgliedern werden beispielsweise nicht weniger geschätzt als die organisationalen Erfahrungswerte langjähriger Mitglieder. Diese Ergebnisse bestätigen drittens den Befund, dass personenbezogene Merkmale wie Engagementdauer und -intensität sowie kommunikative und fachspezifische Kompetenzen von FFF-Aktivist:innen als wertvoll eingeordnet werden. Gleiches gilt für die Vernetzung innerhalb der Bewegung, die wichtiger zu sein scheint als diejenige mit Vertreter:innen von Parteien, NGOs oder Bürger:inneninitiativen. Hinsichtlich des Schauplatzes der öffentlichen Kommunikation kommt bei personenbezogenen Merkmalen des Weiteren hinzu, dass prominentere Mitglieder wie beispielsweise Luisa Neubauer die Agenda von FFF bestimmen (können). Um öffentliche Konflikte zu vermeiden, werden zum Beispiel ihre auf Twitter kommunizierten Positionen von der Bewegung übernommen, da Neubauer von den Medien als Sprachrohr von FFF dargestellt wird. Allerdings machen die Ergebnisse auch an dieser Stelle im Sinne von Freemans (1979) „Democratic Structuring“ deutlich, dass die fehlende basisdemokratische Rückkopplung eher in Effizienz- als Elitismus-Erwägungen begründet ist.

Insgesamt bedarf das Analysegerüst dennoch einer kritischen Reflexion. Zunächst ist wichtig festzuhalten, dass sich die drei Dimensionen in der Natur von Entscheidungsdynamiken überschneiden und entsprechend von Respondents unterschiedlich interpretiert werden können. So konnten beispielsweise im Rahmen der Online-Umfrage von den Teilnehmenden zu den Antworten auf die 22 Fragen keine expliziten Erläuterungen und Motivationen hinzugefügt werden, die Einblicke über die Gründe für die hohen Item-Non-Responses erlaubt hätten. Jenseits der inhaltlichen Interpretation von Umfrageteilnehmer:innen (z. B. Heterogenität, Delegierte/Nicht-Delegierte, Bundes-/lokale Ebene) bleibt ferner unklar, inwiefern die hohe Anzahl von Non-Responses bei jenen Items zeigen, dass bestimmte Fragen eventuell gar nicht oder anders/falsch verstanden wurden. Insbesondere die Tatsache, dass mehrere Fragen in der Online-Befragung unbeantwortet blieben, obwohl die Antwortkategorie „weiß nicht“ angeboten wurde, wirft Fragezeichen für anknüpfende Forschung auf. Sind jene Non-Responses beispielsweise auf das Design der Befragung zurückzuführen oder wurden die Fragen schlichtweg nicht verstanden (z. B. durchaus voraussetzungsvolle Frage nach „informellen Hierarchien“; wenig spezifizierte „Bundesorga“)? Nicht zuletzt hat der Literaturüberblick gezeigt, dass die Begrifflichkeiten um (Wissens‑)Hierarchien und Macht subjektiven Interpretationsspielraum bergen (Willems und Jegers 2012; Sutherland et al. 2014). Der hier verwendete Methodenmix und der Zugang zum Kern der FFF-Szene mithilfe der Citizen Scientists senken zwar das Risiko von *confirmation* und *selection bias* Effekten. Allerdings bedarf es weiterer (v. a. qualitativer) Forschung, um die hier diskutierten Schlussfolgerungen beispielsweise im Hinblick auf die genannten Ausweichkategorien noch stärker empirisch zu unterfüttern.

Die Implikationen für weiterführende Forschung sind dreierlei. Aus Sicht der vergleichenden Politikwissenschaft besteht erstens Erklärungsbedarf, aus welchen Gründen die Überschneidungen bzw. Gleichzeitigkeit von einerseits Basisdemokratie und andererseits Hierarchie das Fortwirken von FFF im nunmehr vierten Jahr des Bestehens scheinbar nicht tangiert. Im Unterschied dazu haben in anderen sozialen Bewegungen im Themenfeld (z. B. ER) und jenseits davon (z. B. OW) im Laufe der Zeit Erosionsprozesse stattgefunden, die unter anderem auch auf asymmetrische Entscheidungsprozesse zurückzuführen sind (Roberts 2012; Welch 2018; Fopp et al. 2021; Friberg 2021). Inwiefern lässt sich im Vergleich dazu die Resilienz von FFF durch die Form und Strukturen interner Entscheidungsprozesse erklären? Was macht FFF dahingehend anders, was macht FFF gar besser? Ebenso besteht dahingehend der Bedarf, den Zusammenhang der eingangs behandelten Generationsperspektive weiter als bislang zu erforschen (Whittier 1997; Coe 2021). Vor diesem Hintergrund bieten sich zweitens Vergleichsstudien an, die die im hiesigen Artikel für den deutschen Kontext erhobenen Daten zu Entscheidungsprozessen bei FFF in ähnlich aktiven Protestpendants beispielsweise in Schweden, Belgien oder Italien untersuchen (Svensson und Wahlström 2021; Giugni und Grasso 2021). Allerdings stellt sich im Hinblick auf komparative Untersuchungsdesigns die grundsätzliche Frage nach den *scope conditions,* bzw. inwiefern der eher stark institutionalisierte Charakter von FFF in Deutschland ein relevanter Einflussfaktor ist, der einen Vergleich erschweren könnte. In diesem Zusammenhang besteht drittens aus wissenschaftstheoretischer und -praxeologischer Hinsicht Forschungsbedarf. Konkret fußt die hiesige Analyse der internen Entscheidungsprozesse auf Interviews, Beobachtungen und Umfragedaten, die ausschließlich von und mit FFF-Mitgliedern durchgeführt wurden. Ohne die Kooperation mit ausgewählten Aktivist:innen im Rahmen des Citizen Science-Projekts wäre ein solcher direkter Zugang in die Bewegung hinein schwer umsetzbar (Mucha et al. 2020). Wie von einem der beteiligten Citizen Scientists treffend beschrieben, wird FFF auf seinen verschiedenen Kanälen von Seiten der Medien und Forschungsanfragen derart „unablässig zugespamt“, dass Mitglieder sukzessive weniger gewillt sind, Umfragen zu streuen oder Interviewpartner:innen zu gewinnen. Vor diesem Hintergrund stellt sich grundsätzlich die Frage nach Alternativen zu Citizen Science-Ansätzen, die in der Lage sind, ähnlich hohe empirische Substanz zu generieren.

## Supplementary Information





