# Supplementary material for: Decision-making and leverage at Fridays for Future—on the role of grassroots democracy, hierarchies, and expertise
Source: Z Politikwiss. 2023 Feb 2:1–29. [Article in German] Online ahead of print. doi: 10.1007/s41358-023-00341-x (PMC9893977; doi:10.1007/s41358-023-00341-x)
Supplement: Supplementary file 1 [file 41358_2023_341_MOESM1_ESM.docx]

**Leitfaden für qualitative Interviews**

| Begrüßung | Vielen Dank, dass Du Dich für ein Gespräch zur Verfügung stellst!   - Vorstellung: Forschungsprojekt an der N.N.-Uni in N.N. - Untersuchung von sozialen Bewegungen am Bsp. FFF - Interview über deine Erfahrungen als FFF-Mitglied - Ca. 30-40 Minuten - Aufzeichnung des Gesprächs, um es später niederschreiben zu können; Anonymisierung; Notizen - Nicht irritieren lassen: Ich schaue auf zwei Bildschirme und mache nebenher Notizen! - Alle Unterlagen können dir später zur Verfügung gestellt werden - Hast du Fragen? Dann können wir loslegen. | | |
| --- | --- | --- | --- |
| Datum: | Alter: | Geschlecht: | Stellung innerhalb von FFF  (wenn zutreffend mehrere):  ☐ Mitglied von OG  Wenn OG, wo:  ☐ Mitglied von AG auf Bundesebene  ☐ Mitglied von AG auf lokaler Ebene  ☐ Delegierte*r  ☐ Sonstige: |
| **Leitfrage** | **Aufrechterhaltungsfragen/Nachfragen** | | **Notizen** |
| 1. **Als erstes würde ich gerne grundsätzlich wissen, wie Treffen bei FFF ablaufen. Wenn du an euer letztes Treffen denkst: Wie geht das so los/wie startet ihr?**   *Inhaltliche Aspekte*   - Technik, Verfahrensweise - Koordination - Bekanntgabe - Abstimmung im Vorhinein | - Wie sieht die Koordination und Bekanntgabe der Termine aus? - Wie läuft die technische Vorbereitung ab? digital/vor Ort? Whatsapp/Zoom? - Wie wird abgestimmt? Handzeichen? Digitale Tools? - Welche Unterschiede gibt es zwischen den einzelnen Ebenen in der Vorbereitung? OG, AG, Deli? | |  |

| **2. Wie kommt es eigentlich zu Entscheidungen? Kannst du dazu was erzählen?**   - 1. Phase: Wie bereitet ihr Treffen/Entscheidungen vor? - 2. Phase: Wenn du an das Treffen von Entscheidungen innerhalb von FFF denkst: Was ist da wichtig? Wer oder was nimmt Einfluss auf Entscheidungen?   *Inhaltliche Aspekte*   - Inhaltliche Vorbereitung - Vorherige Absprachen - Themen-Hierarchien - Machtasymmetrie: Thema vs. Person - Ablauf, Art und Weise der Entscheidungsfindung - Beeinflussung und Lenkung von Entscheidungen - Masse an “Mitläufer*innen”? - Einfluss | 1. *Phase*  - Wie werden Entscheidungen vorbereitet? - Werden vorher alle Beteiligten einer Versammlung informiert? Woher weiß man, worüber man abstimmt? - Wie werden Themen eingebracht? - Wie wird bestimmt, welche Themen diskutiert/entschieden werden? - Gibt es überhaupt vorherige Diskussionen? - Wie laufen diese ab? - Wer koordiniert die Abstimmung? - Sprecht ihr euch vorher ab z.B.?  1. *Phase*  - Werden unterschiedliche Positionen/Meinungen zum Thema vorgestellt oder erläutert? - Wer führt das Wort? Kommt jede*r zu Wort? - Welchen Einfluss haben “Entscheider*innen”/Meinungsführer*innen und “Freundeskreise” auf die Entscheidungsfindung? - Stimmen bestimmte “Freundeskreise” immer gemeinsam ab? - Fallen Dir Beispiele dazu ein, ob Entscheidungen von den Themen abhängen oder von den Personen, die diese einbringen? - Wie können bestimmte Personen oder Gruppen Entscheidungen beeinflussen? Gibt es bestimmte Strategien? Würdest du dich einer Gruppe innerhalb von FFF zuordnen? - Woran liegt es, dass bestimmte Personen sich/ihre Anträge so häufig durchsetzen? Informelle Macht? - Inwieweit hast du selbst schon aktiv versucht, die Entscheidung anderer zu beeinflussen? Wenn ja, wie? Inwiefern wurdest Du aktiv “bearbeitet”? |  |
| --- | --- | --- |
|  |  |  |

| **3. Mich würde interessieren, wie ihr im Nachhinein mit Entscheidungen umgeht: Was passiert eigentlich danach? Wie hast du das erlebt in der Vergangenheit?**  *Inhaltliche Aspekte*   - Korrektur getroffener Entscheidungen - Reflexion - Evaluation getroffener Entscheidungen - Teilhabe und Basisdemokratie - Lernprozesse (operativ und Meta) | - Was ist mit kontroversen Entscheidungen? Verfahren der Evaluation? - Kommt es nach Entscheidungen zu Diskussion/Kritik an diesen Entscheidungen? Sowohl inhaltlich als auch in Hinblick auf die Art der Entscheidungsfindung? - Werden Entscheidungen nach ihrer Umsetzung bewertet? - Wer ist im Nachhinein beteiligt, wenn Entscheidungen neu bewertet werden? - Fallen Dir Beispiele ein, in denen getroffene Entscheidungen im Nachhinein zurückgenommen/ angepasst wurden? - Wären alle Mitglieder an einem solchen Prozess beteiligt? - Wie wird mit Personen umgegangen, die eine andere Meinung vertreten? |  |
| --- | --- | --- |
| **4. Mich interessiert jetzt, wie es dir mit den Entscheidungen geht: Hast du das Gefühl, dass deine Meinung berücksichtigt wird? Wie fühlst du dich dabei? Was ist dir wichtig bei Entscheidungen?**  Auf Dinge eingehen, die uns vorher erzählt wurden: "Du hast ja vorhin gesagt, dass... kannst du das nochmal ausführen?"  *Inhaltliche Aspekte*   - Druck von außen - Rein inhaltliche Aspekte - Meinung anderer - Charismatische/ überzeugende Personen - Eigene Werte | - Wirst du ausreichend gehört in der Bewegung? - Was beeinflusst dich bei Entscheidungen, worauf achtest du? - Welche Rolle spielt Druck aus der Gruppe/vom Umfeld bei deiner Entscheidungsfindung und Abstimmungen? - Sprichst du vorher mit anderen Personen über Entscheidungen? Sind dir andere Meinungen wichtig? - Kommt es (manchmal) zu Kritik an deiner Entscheidung? Wie wird diese geäußert?   Falls “einfaches Mitglied”:   - Wird deine Meinung berücksichtigt von “Entscheider*innen”/höher stehenden Personen?   Falls “höhere Position”:   - Glaubst du, du berücksichtigst die Meinungen von Personen anderer Ebenen ausreichend? |  |
| Willst du noch etwas ergänzen? Ist noch etwas wichtig, was wir noch nicht besprochen haben? | Weltweite Entscheidungen: Globale Streiktermine. Gibt Delegierte auf Europaebene etc., die solche Entscheidungen dann treffen.  Vernetztheit ist bei FFF sehr wichtig, gibt auf jedem Kontinent Gruppen, müssen viele mit einbezogen werden. | |
| Verabschiedung | Vielen Dank, dass du an dem Interview teilgenommen hast!  Du hilfst unserer Forschung damit sehr.  Ich werde jetzt das aufgezeichnete Interview und meine Notizen an das Projektteam schicken und wir kümmern uns dort dann um die Auswertung.  Am Ende des Projekts stellen wir unsere Ergebnisse in einem Projektbericht vor.  Wenn du magst, kannst du uns deine Mail-Adresse geben, dann könnten wir dich nach Abschluss des Forschungsprojekts kontaktieren, um dir diese Ergebnisse zuzuschicken.  Falls du noch Fragen hast, kannst du dich an die Projektleiter*in N.N. wenden.  Du kannst sie über die Mail-Adresse xxx erreichen.  Danke und einen schönen Tag noch! | |
