# Supplementary material for: Decision-making and leverage at Fridays for Future—on the role of grassroots democracy, hierarchies, and expertise
Source: Z Politikwiss. 2023 Feb 2:1–29. [Article in German] Online ahead of print. doi: 10.1007/s41358-023-00341-x (PMC9893977; doi:10.1007/s41358-023-00341-x)
Supplement: Supplementary file 2 [file 41358_2023_341_MOESM2_ESM.docx]

**Fragensammlung für Befragung unter FFF-Aktivist*innen**

Erste Seite – Willkommen

**Herzlich willkommen zu unserer Umfrage!**

Wir bedanken uns für Deine Teilnahme.

Der vorliegende Fragebogen enthält Fragen zu Entscheidungsprozessen der “Fridays For Future”-Bewegung. Es geht dabei um Deine persönlichen Einschätzungen. Es gibt keine richtigen oder falschen Antworten! Deine Antworten werden anonym erfasst.

**Warum erheben und verarbeiten wir Deine Daten**

**Hinweise zum Datenschutz:**

Deine Angaben und Daten werden streng vertraulich behandelt und anonymisiert. Die Ergebnisse werden ausschließlich für diese Studie genutzt. Dies ist eine rein wissenschaftliche Befragung und keine Auftragsforschung. Die Befragung wird von Politikwissenschaftler*innen der Heinrich-Heine-Universität Düsseldorf durchgeführt. Bei Fragen kannst Du uns gerne eine E-Mail an die folgende Adresse schreiben:

xxx

Diese Befragung ist Teil einer Forschungskooperation zwischen Wissenschaftler*innen der Heinrich-Heine-Universität Düsseldorf und Aktivist*innen der “Fridays For Future”-Bewegung in der Region Düsseldorf. Weitere Informationen zum Projekt findest Du [hier](https://www.phil-fak.uni-duesseldorf.de/pw-marschall/forschungsprojekte/fridays-for-future/).

**Wie kannst Du uns kontaktieren**

xxxx

Wenn Du mehr Informationen über die Verarbeitung Deiner personenbezogenen Daten wünschst, bitte auf den folgenden Link klicken.

**Seite 1:**

*V20) Wann hast Du angefangen, Dich bei FFF zu engagieren?* 2018
 im ersten Halbjahr 2019
 im zweiten Halbjahr 2019
 im ersten Halbjahr 2020
 im zweiten Halbjahr 2020
 2021

*V21) Wie aktiv bist Du bei FFF?*

Ich nehme regelmäßig an Treffen von Fridays For Future teil.

Ich nehme ab und zu an Treffen von Fridays For Future teil.

Ich nehme selten an Treffen von Fridays For Future teil.

Ich bin nicht mehr aktiv.

*V24) Wie groß ist die Stadt, in der Du aktiv bist?*

*Bitte gib an, wieviele Einwohner*innen in der Stadt leben, in der Du für FFF aktiv bist.*

mehr als 1.000.000 Einwohner*innen

500.001-1.000.000 Einwohner*innen

100.001-500.000 Einwohner*innen

20.001–100.000 Einwohner*innen

5.001–20.000 Einwohner*innen

2.001–5.000 Einwohner*innen

bis 2.000 Einwohner*innen
 weiß ich nicht

*V26) Bist Du Delegierte*r einer Ortsgruppe?*

[ ] Ja [ ] Nein [ ] nicht mehr [ ] keine Angabe

*V25) Bist Du in einer AG auf der Bundesebene aktiv?*

[ ] Ja [ ] Nein [ ] nicht mehr [ ] keine Angabe

Ab hier sind die Reihenfolge der Fragen auf einer Seite sowieso die Antwortoptionen einer Frage randomisiert.

**Seite 2:**

*V01) Wie zufrieden bist Du mit den Diskussionen in den Gruppen bei Fridays For Future, in denen Du aktiv bist?*

7er-Skala von “unzufrieden” bis “sehr zufrieden” sowie der Kategorie “weiß ich nicht”

*V02) Wenn Du an die Meinungsäußerungen während der letzten Treffen von Fridays For Future denkst, inwiefern stimmst Du den folgenden Aussagen zu oder nicht zu?*

Meine Meinung wird ausreichend berücksichtigt.

Die Meinung der anderen Aktivist*innen wird ausreichend berücksichtigt.

7er-Skala von “stimme gar nicht zu” bis “stimme voll und ganz zu” sowie der Kategorie “weiß ich nicht”

*V03) Entscheidungsprozesse können unterschiedlich ablaufen. Inwiefern stimmst Du den folgenden Aussagen zu oder nicht zu?*

Aktivist*innen werden immer alle an einer Entscheidung beteiligt.

Es sollten immer alle Aktivist*innen an einer Entscheidung beteiligt werden.

Eine gute Entscheidung ist wichtiger als der basisdemokratische Weg dorthin.

7er-Skala von “stimme gar nicht zu” bis “stimme voll und ganz zu” sowie der Kategorie “weiß ich nicht”

**Seite 3:**

*V04) Bei der Fülle von Entscheidungen kann es passieren, dass nicht immer alle Mitglieder daran beteiligt werden. Bitte gib zu jeder der folgenden Aussagen an, inwieweit Du diese zutreffend oder nicht zutreffend findest.*

Es werden nicht immer alle beteiligt, weil…

- ... es zu aufwendig wäre, immer alle einzubinden.
- ... eine Diskussion in kleinen Gruppen fruchtbarer ist als im Plenum.
- ... es wichtig ist, schnell zu einer Entscheidung zu kommen.
- … einige Leute manchmal übersehen werden.
- ... nur diejenigen abstimmen, die Interesse am Thema haben.
- … nicht immer klar ist, dass eine Entscheidung getroffen werden soll.
- Sonstige Gründe: [offenes Antwortfeld]

7er-Skala von “stimme gar nicht zu” bis “stimme voll und ganz zu” sowie der Kategorie “weiß ich nicht”

**Seite 4:**

*V07) Es kann vorkommen, dass bestimmte Personengruppen mehr Einfluss haben als andere. Inwiefern treffen die folgenden Aussagen zu oder nicht zu?*

- In meiner OG gibt es informelle Hierarchien.
- In meiner AG gibt es informelle Hierarchien.
- Auf der Bundesebene gibt es informelle Hierarchien.
- Informelle Hierarchien in der Bewegung entstehen, weil Wissen und Fähigkeiten nicht weitergegeben werden.
- Durch Freundeskreise gibt es innerhalb der Bewegung informelle Hierarchien.

7er-Skala von “trifft gar nicht zu” bis “trifft voll und ganz zu” sowie der Kategorie “weiß ich nicht”

*V08) Persönliche Eigenschaften und Fähigkeiten können den Einfluss einer Person auf die Bewegung bzw. auf Abstimmungen erhöhen. Inwiefern treffen die folgenden Aussagen zu oder nicht zu?*

Ein*e Aktivist*in hat bei Fridays For Future mehr Einfluss wenn er/sie...

- ...mehr Kompetenzen hat.
- ...selbstbewusst auftritt.
- ...schon länger bei FFF aktiv ist.
- ...eine höhere Position in der Bewegung innehat.
- ...freundlich ist.
- ...sehr viel Zeit in die Bewegung investiert.
- ...gut kommunizieren kann.
- ...sich ein Netzwerk innerhalb von FFF aufgebaut hat.
- ...in Parteien, NGOs oder Bürgerinitiativen vernetzt ist.
- ...viele Follower*innen auf Social Media hat.
- Sonstige Faktoren, die den Einfluss erhöhen: [offenes Antwortfeld]

7er-Skala von “trifft gar nicht zu” bis “trifft voll und ganz zu” sowie der Kategorie “weiß ich nicht”

**Seite 5:**

*V09) Was beeinflusst Deine Entscheidungsfindung? Inwiefern sind Dir die folgenden Aspekte wichtig oder unwichtig?*

- Die vorgebrachten Argumente basieren auf wissenschaftlichen Erkenntnissen.
- Der eingebrachte Vorschlag ist umsetzbar.
- Mit der Umsetzung des Vorschlags oder der Aktion können viele Menschen erreicht werden.
- Viele Aktivist*innen können bei der Umsetzung mitmachen.
- Die Entscheidung verspricht eine große mediale Aufmerksamkeit.
- Sonstige Aspekte, die bei Deiner Entscheidungsfindung wichtig sind: [offenes Antwortfeld]

7er-Skala von “unwichtig” bis “sehr wichtig” sowie der Kategorie “weiß ich nicht”

**Seite 6: (nicht randomisiert)**

*V10) Du siehst hier gegensätzliche Aussagen zur Rolle der Wissenschaft für Dich als Aktivist*in. Bitte verorte Dich auf dieser Skala, je nachdem welcher Aussage du eher zustimmst.*

- “Wissenschaftliche Erkenntnisse sind für mich als Aktivist*in für FFF die wichtigsten Leitlinien, um meine Ziele zu erreichen.” (Ursache)     vs.
  “Wissenschaftliche Erkenntnisse sind für mich als Aktivist*in für FFF ausschließlich Instrumente, um meine Ziele zu erreichen.” (Instrument)
- “Wissenschaftliche Erkenntnisse sollten von FFF möglichst präzise als Argument eingesetzt werden, damit sie die Ziele von FFF unterstützen.” (Ursache) vs.
  “Wissenschaftliche Erkenntnisse sollten von FFF auch ungenau eingesetzt werden, wenn sie zum Erfolg von FFF beitragen.” (Instrument)
- “FFF sollte wissenschaftliche Erkenntnisse langfristig in die Gesamtstrategie einplanen.” (strategisch) vs
  “FFF sollte wissenschaftliche Erkenntnisse flexibel und spontan einsetzen.” (situativ)
- “Ich binde wiss. Erkenntnisse als Aktivist*in bei FFF strategisch ein.” (strategisch) vs.
  “Ich setze wiss. Erkenntnisse als Aktivist*in situationsbezogen, je nach Anlass ein.” (situativ)

10er Skala, nach Möglichkeit mit Regler.

**Seite 7:**

*V11) Wenn Dir eine Entscheidung besonders wichtig ist, versuchst Du Dich mit anderen Aktivist*innen vor der Abstimmung abzusprechen?*

7er-Skala von “nie” (1) bis “immer” (7) sowie der Kategorie “weiß ich nicht”

**Seite 9:**

*V13) Es gibt verschiedene Strategien, die bei der Entscheidungsfindung eingesetzt werden können. Wie häufig werden Deiner Einschätzung nach die folgenden Strategien in der Bewegung angewandt?*

- Andere Aktivist*innen schließen sich zusammen, um gemeinsam abzustimmen.
- Andere Aktivist*innen versuchen, mich von etwas zu überzeugen.
- Ich fühle mich von anderen Aktivist*innen unter Druck gesetzt, für oder gegen eine bestimmte Sache zu stimmen.

7er-Skala von “nie” bis “immer” sowie der Kategorie “weiß ich nicht”

**Seite 10:**

*V15) Ein Streitpunkt innerhalb von Fridays For Future ist die Existenz bzw. Nichtexistenz einer “Bundesorga”. Inwiefern treffen die folgenden Aussagen zu oder nicht zu?*

- Es existiert eine “Bundesorga”.
- Die “Bundesorga” hat bei FFF einen großen Einfluss auf Entscheidungen.
- Die tatsächlich gelebten Abläufe bei Entscheidungen weichen von dem basisdemokratischen Selbstverständnis der Bewegung ab.
- Ich bin gut über die Geschehnisse auf Bundesebene informiert.

7er-Skala von “trifft gar nicht zu” bis “trifft voll und ganz zu” sowie der Kategorie “weiß ich nicht”

*V16) Wer hat Deiner Meinung nach am meisten Einfluss auf die Ausrichtung der Bewegung?*

Bitte klicke die Optionen in der Reihenfolge an, die Du für richtig hältst (einflussreichste zuerst).

- Bundesorga
- Einzelne OG
- Einzelne AG
- Delegierte
- Einzelne Personen
- Andere

Als Ranking-Frage abbilden.

**Seite 11:**

*V17) Wie schätzt Du Deinen Einfluss als auch den anderer Aktivist*innen auf die Bewegung ein?*

- Mein Einfluss auf die Bewegung ist ...
- Mein Einfluss in den Gruppen der Bewegung, in denen ich aktiv bin, ist...
- Der Einfluss von Aktivist*innen, die in der Öffentlichkeit stehen, ist auf die Bewegung ...

7er-Skala von “sehr gering” bis “sehr groß” sowie der Kategorie “kann ich nicht einschätzen”

V18) *Stört es Dich, wenn Aktivist*innen, die in der Öffentlichkeit stehen, mehr Einfluss auf Entscheidungen der Bewegung nehmen als weniger prominente Mitglieder?*

*7er-Skala “ja, sehr” bis “nein, überhaupt nicht” sowie der Kategorie “weiß ich nicht”*

*V19) Wie häufig werden die folgenden Entscheidungs- und Abstimmungsformen in Deiner AG/OG eingesetzt?*

In meiner OG/AG wird versucht, einen Konsens herzustellen.

In meiner OG/AG wird eine Mehrheitsentscheidung getroffen.

In meiner AG/OG wird nach Gegenmeinungen gefragt.

Ich fühle mich unter Druck gesetzt, mich der Gruppenmeinung anzupassen.

Entscheidungen werden ohne Abstimmung getroffen, weil ein Konsens angenommen wird.

In meiner AG/OG wird anonym abgestimmt.

In meiner AG/OG wird offen abgestimmt.

7er-Skala von “nie” bis “immer” sowie der Kategorie “weiß ich nicht”

**Seite 12:**

Nun bitten wir Dich noch um zwei Fragen zu Deiner Person.

*V22) Welchem Geschlecht ordnest Du dich zu?*

[ ] Männlich [ ] Weiblich [ ] Divers [ ] keine Angabe

*V23) Wie alt bist Du? (Auf 2 Kästchen begrenzter Freitext)*

Bitte gib Dein Alter in Jahren an.

Letzte Seite der Umfrage

**Du hast es geschafft!**

Hast Du noch Anmerkungen zu der Befragung?

[ Freies, offenes Feld ]

End-Seite

**Wir danken Dir herzlich für die Teilnahme an der Befragung und wünschen Dir alles Gute.**
